# Patterns of shade plant diversity in four agroforestry systems across Central America: a meta-analysis

**DOI:** 10.1038/s41598-023-35578-7

**Published:** 2023-05-26

**Authors:** M. Jimena Esquivel, Sergio Vilchez-Mendoza, Celia A. Harvey, Mayra A. Ospina, Eduardo Somarriba, Olivier Deheuvels, Elias de M. Virginio Filho, Jeremy Haggar, Guillermo Detlefsen, Carlos Cerdan, Fernando Casanoves, Jenny C. Ordoñez

**Affiliations:** 1 CATIE (Centro Agronómico Tropical de Investigación y Enseñanza), 30501 Turrialba, Costa Rica; 2 Animal Production System Group (APS), Department of Animal Sciences, Wageningen University, 6708 WD Wageningen, The Netherlands; 3 Institute of Environmental Sciences (CML), Environmental Biology, Leiden University, 2333 CC Leiden, The Netherlands; 4 Centro para la Investigación en Sistemas Sostenibles de Producción Agropecuaria – CIPAV, Cra 25 # 6-62, Cali, Colombia; 5 Monteverde Institute, Apdo.69-5655, Monteverde, Puntarenas, Costa Rica; 6 CIRAD, UMR ABSys, Univ Montpellier, CIHEAM-IAMM, CIRAD, INRAE, Institut Agro, 34398 Montpellier, France; 7 CIRAD, UMR ABSys, 10126 Santo Domingo, República Dominicana; 8 Natural Resources Institute, Faculty of Engineering and Science, University of Greenwich, Medway Campus, Central Avenue Chatham Maritime, Kent, ME4 4TB UK; 9 Facultad de Ciencias Agrícolas, Universidad Veracruzana,, Lomas del Estadio S/N, Col. Zona Universitaria, C.P. 91000 Xalapa, Veracruz, México; 10 Grupo de Investigación en Agroecosistemas y Conservación en Bosques Amazónicos (GAIA), Universidad de La Amazonia, Florencia, Colombia; 11 Universidad de Las Américas, Carrera de Ingeniería Agroindustrial, Udlapark, Quito, Ecuador; 12 Word Agroforestry Centre (ICRAF)-Latin America. DID, CATIE 7170, Turrialba, 30501 Costa Rica

**Keywords:** Biodiversity, Forest ecology, Agroecology, Conservation biology

## Abstract

Agroforestry systems can potentially increase tree diversity within agricultural landscapes, but to date, there is little understanding of the patterns of shade plant diversity within different agroforestry systems (AFS) at large spatial scales. Using compiled plant inventory data (from 23 sources, 2517 plots, and 148,255 individuals) encompassing four AFS (shaded coffee; shaded cocoa; dispersed trees on pastures; and live fences) across six countries in Central America we estimated different metrics of diversity to assess the conservation value of different AFS for shade plants. 458 shade plant species were recorded across the four agroforestry systems. Primary forest species accounted for 28% of the shade species recorded, but only 6% of the recorded individuals. No single AFS was consistently the most diverse across countries when considering rarefied species richness. Trees on pastures can potentially reach a similar species richness as cocoa and coffee systems but require sampled areas 7–30 times larger. In terms of composition, 29 species were shared across the agroforestry systems in different countries, illustrating the strong selection pressure of farmers for species that provide timber, firewood, and fruit. Our study highlights the potential contribution and limitations of different AFS for tree diversity conservation within agricultural landscapes.

## Introduction

The future of biodiversity conservation in the highly modified landscapes of Central America will depend, in large measure, on how agricultural landscapes are managed. Central America is a region of high conservation value due to its unique mixture of flora and fauna from the Nearctic and the Neotropics and includes some of the most biodiverse ecosystems in the world^1,2^. Most land in Central America was originally forested, however, this forest has undergone a continuous transformation since pre-Columbian times^3^. Currently, agricultural land in Central America covers ~ 51% of the total land area while natural forests cover only 36% (data from 2010 to 2016^4^).

While the remaining forests are critical for biodiversity conservation in region^5^, the presence of shade plants on farms can also help to conserve plant and animal diversity^6,7^. By ‘shade plant’, we refer to woody vegetation including trees and shrubs; large monocots (i.e. *Musaceae* and *Arecaceae*), and in a few cases large tropical herbs and vines (i.e. *Urera laciniata*, *Adenopodia patens*) that are used to provide shade to crops and pastures. Diverse agroforestry systems (such as coffee and cocoa agroforestry systems or silvopastoral systems including dispersed trees in pastures and live fences) can help conserve biodiversity by increasing plant density and diversity, providing habitat and resources for wild species, and enhancing the functional connectivity of agricultural landscapes^6,8–11^. For example, trees in agricultural landscapes can create biological corridors among natural reserves and/or remaining forest fragments, facilitating the colonization, movement, and survival of forest species^12,13^.

The establishment of diverse agroforestry systems (hereafter abbreviated as ‘AFS’) is considered one of the most promising options for maintaining tree cover and conserving diversity and connectivity in agricultural landscapes while maintaining agricultural production^8^. Multiple studies have shown that, even if the local conservation value of AFS is generally much lower than that of native forests^5,14,15^, AFS can harbor a high richness of terrestrial and epiphytic plants, fungi, birds, mammals, reptiles, amphibians, and insects^16–20^. Within AFS, farmers plant, retain from the natural regeneration or simply tolerate^21^ multipurpose plants that provide food, traditional medicines, timber, firewood, and important ecosystem services^22–25^. However, since the natural regeneration of primary forest tree species is often limited within agricultural fields^18,24,26^, over the long term, plant diversity within agricultural landscapes can decline if remnant tree species are not replaced through adequate management of natural regeneration^27^.

Despite growing interest in agroforestry, there is still limited information on the patterns of plant abundance, density, and diversity across different AFS. To date, patterns of plant diversity in AFS have been mainly assessed through local studies focused on a particular type of AFS in one or a few sites^9,28–31^ that either considered how diversity changes across time within a particular AFS^32^ or lumped different AFS in a single class^5^. However, AFS are highly variable in terms of their spatial and temporal arrangements, composition (different species of crops, woody plants, and animals), and management practices. As such, comparisons of tree diversity across multiple types of AFS and multiple countries are still needed.

The objective of this article is to characterize patterns of shade plant diversity in four agroforestry systems in Central America, including shaded coffee systems, shaded cocoa systems, dispersed trees in pastures, and live fences, to better understand the ability of AFS to contribute to tree diversity conservation efforts. More specifically we address the following research questions related to the conservation value of agroforestry systems:

A) Do agroforestry canopies harbor woody species of conservation value? For this, we assess the patterns of abundance of shade plants (considering growth forms, whether species are native or exotic, and successional guilds) in different AFS in Central America.
B) How diverse are the canopies of different agroforestry systems? For this, we compare the expected number of species under different agroforestry systems to assess whether there are systems which consistently have higher species richness irrespective of the country where they are sampled.
C) How similar are AFS in Central America in terms of species composition? To explore this, we assess to what extent different AFS overlap in shade plant species composition and assess which are the most common shade species found in agroforestry systems.

Central America is one of the regions with the largest tree cover outside forests^33^ and is home to human communities where agroforestry practice is embedded in cultural practices^34^. For instance, the production of coffee and cocoa in the region is almost entirely managed under shade^29,35^, although levels of shade vary significantly across countries and landscapes^36^. Trees in pastures and live fences are a conspicuous feature of pastures, providing numerous benefits for ecosystem services and livelihoods^30^. The maintenance and expansion of agroforestry are encouraged by national governments, coffee institutes (e.g., IHCAFE, ICAFE), development programs (e.g., GIZ), and research organizations (e.g., CATIE, CIAT, CIRAD, FHIA, ICRAF) active in the region. In this context, understanding how agroforestry systems contribute to the conservation of shade plant diversity is of prime importance. The results of this study offer information about both the potential windows of opportunity and limitations of leveraging shade plants on farms for biodiversity conservation.

## Results

### Conservation value of different agroforestry systems

The 2,517 plots and 148,255 individuals analyzed corresponded to four AFS: cocoa agroforestry systems (COCOA-AFS), coffee agroforestry systems (COFFEE-AFS), dispersed trees in pastures (DTP), and live fences (LF) across six countries: Panama (PAN), Costa Rica (CRI), Nicaragua (NIC), Honduras (HND), Guatemala (GTM) and Belize (BEL)*.* Across the dataset, 68.8% of individuals were identified to species level, 26.6% were identified to the genus level, 4.4% were identified only with vernacular names, and 0.3% were unidentified. Of the individuals identified at species and genus level (n = 141,357 individuals), 72.8% were trees, 22.5% were plantains or bananas (*Musaceae*), 4.4% were palms (11 species in *Arecaceae*), and 0.3% corresponded to small shrubs (19 species), woody monocots, large herbs, and climbers (Appendix C). Across 20 AFS × country combinations, we found a total of 458 shade species (plus 65 only identified to genus level) corresponding to 276 genera and 81 families. Of these, 49 corresponded to exotic species (plus two identified to genus level; Appendix C). Exotic species accounted for 26% of the total abundance (mainly due to the abundance of *Musaceae*). The ten most abundant families (in order of importance) were *Fabaceae* (24.6% of individuals)*, Musaceae* (22.5%)*, Boraginaceae* (11.9%)*, Malvaceae* (6.5%)*, Bignoniaceae* (4.5%)*, Arecaceae* (4.4%)*, Meliaceae* (3.6%)*, Rutaceae* (2.9%)*, Burseraceae* (2.5%) and *Anacardiaceae* (2.2%), which together accounted for 85.6% of all individuals. There were 18 families represented by ten or fewer individuals in the whole sample (Appendix D).

Across all AFS, the ten most abundant species accounted for 61.2% of all individuals. These species were (in order of abundance): *Musa* spp. (22.5%)*, Cordia alliodora* (11.5%)*, Gliricidia sepium* (9.0%)*, Guazuma ulmifolia* (4.5%)*, Tabebuia rosea* (3.3%)*, Cedrela odorata* (2.6%)*, Bursera simaruba* (2.4%)*, Inga* spp. (2.0%)*, Bactris gasipaes* (1.8%)*,* and *Citrus maxima* (1.5%). At the tail end of the abundance distribution, 50 shade species had only one individual, and 27 species had two individuals in the whole sample (plus another 20 species that were only identified to genus level). In terms of frequency, the average number of plots in which a species occurred was 29.8 plots (out of 2,517). The species with the highest frequency of occurrence was *Cordia alliodora* (found in 1,042 plots out of 2,517). Other frequent species included *Cedrela odorata* (present in 649 plots)*, Gliricidia sepium* (574 plots)*, Tabebuia rosea* (522 plots), *and Guazuma ulmifolia* (516 plots). 108 shade species were present only in one plot (including 21 which were identified only to genus level).

Successional guild information was retrieved for 382 woody species (391 native, trees, and shrubs species); there were nine species for which we could not retrieve any information. Of these 382 native species, 28% corresponded to intermediate and primary forest species, 37% were species from secondary forests and 10% were species whose forest successional stage was unclear (see Appendix C). The remaining 25% corresponded to species typical of open and agricultural areas and generalists (found both inside and outside forests). In terms of abundance, trees from open and agricultural areas and generalists accounted for most of the individuals (70%). Secondary forest species accounted for 21% of all individuals, while intermediate and primary forest species represented only 6% of all individuals. Moreover, intermediate, and primary forest species were present in 743 plots (out of 2,517) and, in the plots where they were present there was a median of 2 individuals per plot. Fifty species were singletons, meaning that they were represented by only one individual in the whole dataset. Of the 50 singleton species, 50% corresponded to intermediate and primary forest species, 20% to secondary forest species, and 22% corresponded to trees that occur outside forests or generalists that occur in open areas and forests. The remaining 8% corresponded to forest species whose forest successional status is unclear.

### How diverse are the assessed AFS?

**Figure 1:**
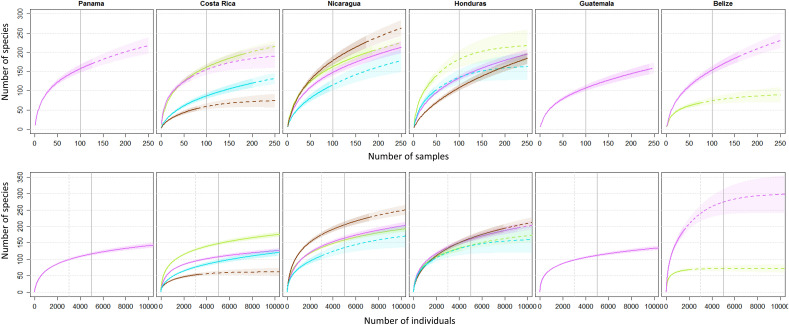
Rarefaction and extrapolation curves with Hill numbers (qD, for species richness where q = 0) per samples (sites), (upper panel) and per individuals (lower panel), for six countries and four agroforestry systems: COCOA-AFS (purple); COFFEE-AFS (brown); DTP (green) and LF (turquoise). Perpendicular lines represent the level at which comparisons for richness were made per samples: continuous grey line for comparisons at S100 = 100 samples and i5000 = 5 000 individuals and dashed grey line for comparisons at S100 = 100 samples and i3000 = 3 000 individuals, see methods for more details.

We assessed expected species numbers (here onwards referred to as ‘richness’) using rarefaction curves based on samples (plots) and individuals (Fig. 1, Appendix E). Rarefaction curves of each AFS were compared within each country at 100 samples (R_s100_) and 5,000 individuals (R_i5000_). There was no clear pattern of one AFS being consistently more diverse than another across different countries, as species richness for each AFS depended strongly on the country of study (Fig. 1). In Costa Rica, considering species richness estimates per sample, DTP (R_s100_ = 161) and COCOA-AFS (R_s100_ = 155, See Fig. 1) had the highest species richness and were not different from each other. Nevertheless, DTP estimates were obtained with sampled areas ~ 30 times larger than those in COCOA-AFS in CRI (See Table 1). The COFFEE-AFS in CRI was the system with the lowest species richness (R_s100_ = 59), even below LF (R_s100_ = 86, Fig. 1). When considering the number of species per individuals, all systems were significantly different from each other. DTP had the highest richness in CRI (R_i5000_ = 147) followed by COCOA-AFS (R_i5000_ = 107), LF (R_i5000_ = 92), and finally COFFEE-AFS (R_i5000_ = 58).

**Table 1:** Number of plots, plot size descriptive statistics, and total area sampled per agroforestry system (DTP = dispersed trees on pastures; COCOA-AFS = cocoa agroforestry; COFFEE-AFS = coffee agroforestry and LF = live fences) and country for the 2,517 plots selected for analysis (see methods).

	DTP		COCOA-AFS		COFFEE-AFS		LF		COUNTRY TOTAL	
Country	Number plots	Sampled area (ha)	Number plots	Sampled area (ha)	Number plots	Sampled area (ha)	Number plots	Sampled area (ha)	Number of plots	Sampled area (ha)
Panama	5	0.5	127	88.1			19	0.4	151	89.0
Costa Rica	180	1,150.9	67	41.3	81	8.3	202	109.1	530	1,309.7
Nicaragua	188	515.5	272	68.2	174	41.8	93	33.6	727	659.1
Honduras	47	43.5	249	89.3	272	25.6	46	43.0	614	201.4
Guatemala			240	68.5	19	0.5			259	68.9
Belize	75	72.5	159	15.9			2	0.2	236	88.5
AFS TOTAL	495	1,782.9	1114	371.3	546	76.1	362	186.2	2,517	2,416.6
Plot size statistics (ha)										
Range	0.04–161.0		0.07–9.8		0.03–0.5		0.01–1.6			
Median	1.00		0.10		0.10		0.43			
Average	3.60		0.33		0.14		0.51			

In Nicaragua, considering estimates per sample, COFFEE-AFS (R_S100_ = 179) had the highest species richness followed by DTP (R_S100_ = 163) and COCOA-AFS (R_S100_ = 148). DTP reached these levels of species richness with a total sampled area ~ 7.5 times larger than COCOA-AFS and ~ 12 times larger than COFFEE-AFS. LF was the system with the lowest species richness (R_S100_ = 115). Considering the number of species per 5,000 individuals, COFFEE-AFS (R_i5000_ = 205) had the highest species richness. Species richness in COFFEE-AFS was significantly different from both COCOA-AFS (R_i5000_ = 164) and DTP (R_i5000_ = 158) which were not different from each other. Again, the least diverse system was LF (R_i5000_ = 135).

In Honduras, considering estimates per 100 samples, DTP (R_s100_ = 182) had the highest species richness. Unlike Costa Rica and Nicaragua, these results were obtained in HND with a total sampled area of 43.5 ha for DTP, a value that is between the total sampled area of COCOA-AFS (89.3 ha) and COFFEE-AFS (25.6 ha, Table 1). Live fences (R_s100_ = 135) and COCOA-AFS (R_s100_ = 133) had intermediate species richness and were not different from each other. Finally, COFFEE-AFS (R_s100_ = 108) had the lowest species richness; this was also the AFS with the lowest total sampled area in this country. In terms of individuals, there were no significant differences among the four systems. COCOA-AFS had the highest species richness (R_i3000_ = 136) followed closely by COFFEE-AFS (R_i3000_ = 133), DTP (R_i3000_ = 122), and finally LF (R_i3000_ = 121). In Panama, Guatemala, and Belize, comparisons of species richness across different AFS were not possible due to the lack of sufficient data.

### Similarities and differences in the composition of different AFS

**Figure 2:**
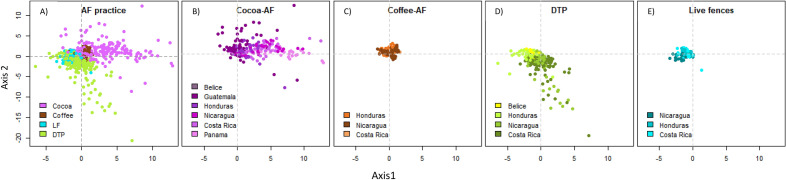
Shade species scores across Axis 1 and 2, estimated using non-metric multidimensional scaling (NMDS). (**A**) Groups with different colors represent 4 agroforestry system: COCOA-AFS (purple); COFFEE-AFS (brown); DTP (green) and LF (turquoise), (**B**–**E**) Shade species scores, per agroforestry system showing country groups with different colors (for each country, see figure inner legends). Countries have been ordered from north to south to highlight potential differences in composition by latitudinal gradient.

The first main axis resulting from the non-metric multidimensional scaling analyses (NMDS) explained 0.69 of the spatial distribution of species composition across the 18 AFS × country combinations; while the second axis explained 0.29 (Fig. 2), with a stress value of 11.26% indicating a reasonable ordination^37^. There was a clear difference in species composition among AFS particularly for COCOA-AFS that were positioned mostly along Axis 1 and DTP systems that were positioned mostly along Axis 2 (Fig. 2). The distances between the clusters COCOA-AFS and DTP systems were larger in the NMDS bi-dimensional space, reflecting their differences in plant composition. Live fences and COFFEE-AFS formed well-defined clusters with a large overlap around the center of both axes, reflecting the similarity in species composition among these AFS.

**Figure 3:**
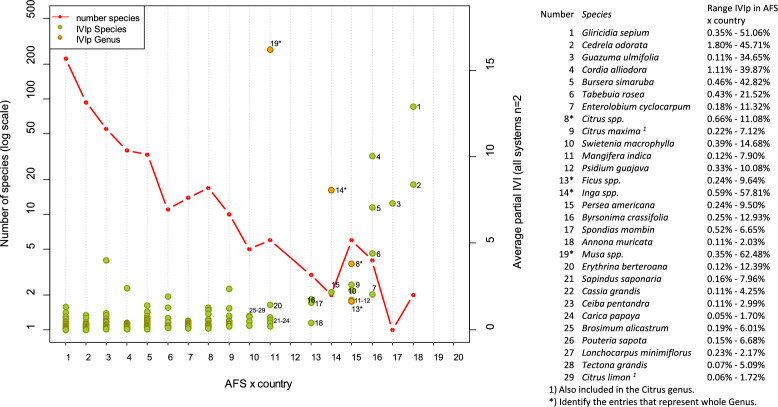
Number of species and average partial IVI partial values (*IVIp*) per possible AFS × country combinations. The X-axis represents the number of AFS × country combinations possible. Since data for the four AFS was not available in all six countries, n AFS × country = 20 in this study. Red dotted line: number of species (plotted in a log10 scale, left Y axis) that were present in different AFS × country combinations. Dots in light green represent *IVIp* for each species across all possible AFS × country combinations. The dots in orange represent *IVIp* for four genus with the highest *IVIp*. Numbers are used to identify the species and genus that were present in at least 10 AFS × country combinations, for each entry also the range of the species IVI partial values is presented in a table. For instance, there were > 200 species present in only one AFS × country combination, while there were two species (*Gliricidia sepium* and *Cedrela odorata*) present in 18 AFS × country combinations. *Gliricidia sepium* had an *IVIp* of 13% (with IVIp values in different AFS × country combinations that range between 0.35 and 51.06%).

The analysis using the Partial Importance Value Index (IVIp) of species in each of the 20 AFS × country combinations and the Average-IVIp $\left( {\underline{{IVI_{p} }} } \right)$ of a species across all AFS × country combinations allowed us to explore patterns of species composition. Twenty-nine species were present across more than 10 AFS × country combinations; however, these species were not necessarily the most dominant (Fig. 3). For instance, G*liricidia sepium* ($\underline{{IVI_{p} }}$ = 12.9% ± 4.1%) and *Cedrela odorata* ($\underline{{IVI_{p} }}$ = 8.4% ± 2.4%) were present in 18 out of 20 AFS × country combinations, but their IVIp values ranged from 0.35% up to 51% showing that they were always present but had low abundances in some AFS × country combinations. Other common species were present in 14 to 17 of the AFS × country combinations, including *Guazuma ulmifolia, Cordia alliodora, Bursera simaruba, Tabebuia rosea, Enterolobium cyclocarpum, Citrus maxima, Swietenia macrophylla, Mangifera indica, Psidium guajava,* and *Persea americana (see* Fig. 3 for species IVIp values). The most common genera were *Citrus* spp. (n AFS × country = 15), *Ficus* spp. (n AFS = 15), *Inga* spp. (n AFS = 14) and *Musa* spp. (n AFS = 11). The *Musa* genera had the highest $\underline{{IVI_{p} }}$ = 16.2% ± 6.2%, due to their high densities in COFFEE-AFS and COCOA-AFS. At the tail end, 193 species were present in only one AFS and another 81 species were present in two AFS × country combinations (entries identified only to genus level were not counted).

## Discussion

### Shade plant diversity in agroforestry systems

Overall, our study shows that Central American AFS retain a significant number of trees within agricultural fields and pastures. Across the AFS studied, we recorded 458 shade plant species occurring in shade canopies. This is a low number of species if compared to the high diversity found in tropical forests and moderate if compared to similar studies in other AFS (120–424 species) sampled in smaller areas^27^. The tree assemblage in AFS is highly modified in comparison to natural forests but most of the tree species were native. Most of the trees in the AFS studied stem from natural regeneration^21^ rather than the introduction of exotic species by active planting, a trend more common in other regions^38^. The presence of these native trees within agricultural lands can improve landscape structure and serve as biological corridors for plants and other taxa such as mammals, frugivorous birds, and insects^14,15,17,20,39^, thereby enhancing the conservation value of agricultural landscapes.

However, our study also suggests that the tree composition within AFS is highly modified compared to that of intact forest. In all the AFS the species composition of the shade canopy was skewed towards secondary forest species and tree species that are useful for farmers. For example, although primary forest species accounted for 28% of the woody species recorded, they represented only 6% of the recorded individuals. In contrast, species from open areas and agricultural areas accounted for only 25% of the species, but 70% of all individuals. As such, the value of the AFS for the conservation of forest plant species is clearly much lower than that of natural forests.

At the same time, the low abundance of late-successional forest species illustrates the challenges for the conservation of diversity in the long term. Many of the late-successional forest species were represented by a median of two individuals in the plots where they were present, and 25 late-successional species (out of 50) were represented by only a single tree in the whole dataset. For these species, the loss of solitary trees could have significant negative impacts at the landscape level. Whether these tree species present in low abundance will persist in the AFS over the long term will depend on species-specific reproductive traits such as mating and breeding systems^40,41^, seed dispersal syndromes^42^ and the presence of pollinators^43^, as well as farm management practices (such as weeding, shade pruning, grazing regimes). The use of fire, non-selective weed control, and uncontrolled grazing contribute to the reduction of tree cover and can have profound negative impacts on trees present in low densities^18,30^.

### How similar is shade tree diversity across agroforestry systems in Central America?

Our results suggest that there is no single AFS which consistently was the most species-rich across all countries. For instance, the highest species richness in Costa Rica was found in DTP and COCOA-AFS, in Nicaragua in COFFEE-AFS, and Honduras in DTP systems. Conversely, the least diverse systems were COFFEE-AFS in Costa Rica and Honduras and LF in Nicaragua.

Many of the differences in shade tree diversity across AFS likely reflect differences in crop and shade management practices. In COFFEE-AFS, shade tree diversity is lower in countries that have intensified coffee production and simplified shade composition^16,36^. For instance, coffee production systems in Costa Rica and Honduras had consistently lower species richness than silvopastures or cocoa systems in the same countries. Coffee production systems in Costa Rica are generally considered the most intensified in the region^44,45^, with farmers managing a simplified shade canopy of useful trees such as *Erythrina* spp. and *Musaceae*. In Honduras, extension services have also traditionally promoted a model of coffee production under a simplified canopy of Inga species planted at low densities (8–10 m apart) and pruning of tree branches, leaving a thin canopy 2–3 m above the coffee plants (Somarriba, personal communication). In these systems, the native forest species are almost completely removed, and tree diversity is low^16^. In contrast, in Nicaragua, coffee is produced under commercial polycultures with a shade of mainly *Musaceae* and *Erythrina* spp., but also under more traditional systems with more diversified shade^29,46^.

Second, our results suggest that the species richness of DTP can reach that of COFFEE-AFS and COCOA-AFS but requires a sample area that is ~ 10 to 30 times larger than that of COFFEE-AFS or COCOA-AFS. These results indicate that despite their low species density, the species turnover in DTP is high probably due to a high species diversity per individual and variable species composition across DTP plots. Moreover, the diversity patterns of DTP could have important effects at landscape scales since pastures occupy between ~ 29–69% of agricultural land in different countries of Central America while coffee and cocoa (mainly planted under AFS) occupy between 0.5 and 9.6%^4^ (the range refers to each crop individually). Given the large extension of pastures with low tree densities, relatively small changes (increasing or decreasing) in tree cover could have profound impacts at the landscape scale.

Finally, our results from the analysis of abundance and IVIp values, and the high similarity shown by clustering around the middle of the Axis 1 and 2 of the NMDS analysis for all the systems, suggest that the composition of all AFS is somewhat uniform across systems and countries. There is a reduced set of species that is common across the four AFS and the Central American region. This set of common trees includes timber species (*C. odorata, C. alliodora*), fruit trees (*Musa* spp.*, Citrus* spp.), fodder trees (*E*. *cyclocarpum, G. sepium, Bursera simaruba*), and N-fixing trees (*Inga* spp.*, Erythrina* spp.) which have rapid growth, prolific regeneration and are easy to propagate by seeds (e.g. *G. ulmifolia, B. crassifolia, P.guajava*) and cuttings (*Tabebuia rosea*, *G. sepium, Erythrina* spp.). Farmers actively select and retain (and sometimes plant) these tree species because of their value for farm production and their contribution to family livelihoods, in terms of avoided purchases, safety nets, and food and nutritional security^22,23^. Moreover, farmers only use part of the total timber, firewood, and fruit resources available in their farms. For instance, in Nicaragua, farmers report a modest timber harvest ratio at the farm level: < 1 tree ha^−1^ year^−1^ ^47^, and 45–60% of fruits are lost (not used or sold) in coffee agroforestry systems in Guatemala and Peru^23^. Therefore, it should be possible to increase or maintain the tree cover of species of conservation value without reducing the benefits that farmers already derive from their trees.

### Implications for tree diversity conservation in Central America

Our study provides four key insights for tree conservation efforts in the region. First, our study suggests that AFS can play a potentially important role in efforts to conserve tree diversity in the region. Although the AFS studied have a highly modified flora (compared to natural forests), they still harbor a rich diversity of native tree species, retaining more than 480 tree species. While many of the trees within these systems are early successional species, AFS also retain some forest and late successional species which are important for both plant and animal conservation efforts. In addition, shade trees within the AFS provide critical habitats, connectivity, and resources for the conservation of wildlife within agricultural landscapes^6,10^ and enhance the natural regeneration of trees within the agricultural matrix^18^. Programs focused on trees on farms could make important contributions to the maintenance and restoration of natural forests across agricultural landscapes, while also offering farmers ways to improve their farming systems and livelihoods. To ensure long-term success, these efforts to conserve biodiversity within AFS should be part of larger, landscape-level efforts that include the conservation and restoration of forests and other natural ecosystems (i.e., through protected areas, community-managed forests, and related measures).

Second, our study highlights the important effect of farm management and species selection on shade tree diversity within AFS, and the need for active farmer participation in conservation efforts. Programs that seek to conserve biodiversity across Central America’s agricultural landscapes could further enhance the shade tree diversity within AFS by encouraging farmers to retain a greater diversity of tree species and individuals within their agricultural fields and pastures; to diversify the set of tree species that they actively plant and manage as shade; and to increase the share of species of conservation values. To encourage greater conservation action, conservation programs could potentially support farmers to access payments for ecosystem services, carbon credits, targeted premiums for species of conservation value, certification processes, or other types of rewards or compensation^36,48,49^. In addition, these programs could enhance farmer access to information and training on timber production, species selection, tree management, and related aspects. Another important aspect is the need to improve farmer access to tree germplasm for native trees, for instance through capacity building to develop local nurseries or free nurseries supported by NGOs or government programs that make it easy for farmers to access germplasm of native tree species^50^.

Third, our study highlights the need to pay more attention to shade species diversity and management within the region’s vast expanses of pastures. Given the large areas that DTP systems occupy in the agricultural matrix, small changes in tree cover or species turnover could have large impacts at the landscape scale. In recent years, the policy agenda has shifted towards intensifying existing pasture areas, while allowing areas of unproductive pastureland to restore forests^51,52^. Such policy changes could become an opportunity for biodiversity conservation if efforts to increase productivity in these systems go hand in hand with the implementation of practices and incentives to maintain a cover of useful trees and increase tree cover of high conservation value species within cattle production areas^53^. On the other hand, if intensification efforts discourage the use of silvopastoral systems, this could significantly reduce biodiversity conservation and ecosystem services within agricultural landscapes.

Finally, our study highlights the need for greater standardization of methods for characterizing and monitoring the patterns of shade tree abundance and diversity within AFS. Although shade trees are a conspicuous feature of agricultural landscapes, the methods for measuring tree abundance, species richness, and diversity are not standardized, and this limits our ability to assess the conservation value of trees and track changes over time. There is a need for standardized methodologies for assessing the patterns of tree diversity on farms that are cost-effective, easy to apply, scientifically robust, and comparable. The adoption and use of standardized methodologies would allow us to assess the status and changes in tree resources within agricultural landscapes and would provide valuable information for decision-making for conservation efforts.

## Methods

### Data compilation, handling, and selection

To assess the abundance, species richness, and diversity of shade plants in different AFS, we compiled data from twenty-three sources that had plant inventory or census information from AFS in Central America (Appendix A). Sources included individual studies and entire research projects developed between 2003 -2011 by researchers from the Centro Agronómico Tropical de Investigación y Enseñanza (CATIE). The original data compilation included plant inventories from 3,478 agroforestry plots. After an initial screening, we excluded duplicates, plots with no area information, plots with only one shade plant species, and AFS that were recorded in only one country. The final database included 2,546 plots and 158,247 plants (Appendix A), with data from four AFS: cocoa agroforestry systems (COCOA-AFS), coffee agroforestry systems (COFFEE-AFS), dispersed trees in pastures (DTP) and live fences (LF). The database included information from six countries: Panama (PAN), Costa Rica (CRI), Nicaragua (NIC), Honduras (HND), Guatemala (GTM), and Belize (BEL)*.* The scientific names of all plants were updated to the latest accepted names using World Flora Online (http://www.worldfloraonline.org/, last visited 14 April 2021). We also checked vernacular names for consistency and spelling errors.

As the objective of the study was to characterize the diversity of the shade plants in different AFS, we defined what elements of the vegetation could be considered “shade plants” and updated the databases accordingly. For this study, we defined “shade plants” as those that are regularly intercropped with either coffee (*Coffea arabica* L.) or cocoa (*Theobroma cacao* L.); or are interspersed on pastures (*Poaceae*); or are planted in lines between agricultural fields. Shade plants generally occupy a higher stratum in the canopy than the main crop and generally have perennial lifespans. Our definition of shade plants includes woody vegetation (trees and shrubs), large monocots (i.e., *Musaceae* and *Arecaceae*), and in a few cases large tropical herbs and vines (i.e., *Urera laciniata, Adenopodia patens*). The full list of species was assessed to identify crop and shade species, and all data entries (individuals within plots) that corresponded to crops such as coffee, cocoa, maize (*Zea mays*), and cassava (*Manihot esculenta,*), (in total 1,111 individuals across the 2546 plots) were eliminated from the inventories.

Before analyzing the data, we carried out basic descriptive statistical analyses to identify potential issues in the structure of the data that could affect the results of the diversity analysis. First, we assessed the proportion of individuals identified to vernacular and scientific names vs. non-identified (NO-ID) individuals. Of the 2,546 plots, only 144 plots had some NO-ID individuals. Across the whole dataset (n = 157,136 shade individuals), 95.6% were individuals identified with scientific names (to genus or species level), 4.2% were identified with vernacular names and 0.2% were NO-ID. The management of these NO-ID individuals for the diversity analysis is discussed further in the description of the methods of each diversity index (see below).

Second, we assessed the distribution of plot sizes, abundance, and density (individuals ha^−1^) of shade species per AFS to identify the presence of extreme values. For the specific case of LF systems which were sampled in lines rather than plots, we assumed a lateral width of 2 m (1 m at each side of the fence) to transform fence length to plot area; thus if a fence was sampled in a line of 50 m, the plot area corresponded to 100 m^2^. There were differences among the studies in terms of the sampling efforts, number of plots, and sampled areas. For instance, in some cases the studies applied inventories in sample plots (e.g., fixed area plots ranging in size from 100 to 10,000 m^2^) within the farmer plots (the crop management units within farms); in other studies, researchers conducted a full census of all species within farmer plots (with variable areas) or a full census of all trees within the farm. In general, data for plot sizes, abundances, and density were strongly right-skewed. In terms of plot size, larger plots were inventoried in DTP, while smaller plots were more common in COFFEE-AFS and COCOA-AFS (Table 1). Moreover, there were a few plots with very low or high shade densities (see Appendix B) but there was no other information available in the original sources to discern whether these values were correct or not. To avoid problems with extreme values of shade densities, plots with density values that fell below the 0.5 percentile or above the 99.5 percentile of each agroforestry system were excluded from the dataset (n = 29 plots). After this selection, the final dataset used for analysis encompassed 2,517 plots and 148,255 individuals, covering 20 AFS × country combinations (Table 1) and a total sampling area of 2416.6 ha (of which 73.8% corresponded to DTP, 15.4% to COCOA-AFS, 7.7% to LF, and 3.2% to COFFEE-AFS). Across the six countries assessed, Costa Rica, Nicaragua, and Honduras had information on all four AFS (Table 1). In contrast, in Panama, Guatemala, and Belize data was available for only one or two AFS.

### Data analysis

#### General patterns of abundance

For all species identified to species level (n ~ 390), we derived information regarding taxonomic families, species origin (native or non-native to the Central America region, with ‘non-native’ species hereafter referred to as “exotics”), growth form and successional guilds from expert knowledge of the authors (for the most well-known species in the region), online databases (e.g. World Flora Online and GBIF www.gbif.org, last visited in April 2021), peer-reviewed publications and grey literature (e.g., theses, reports and other publications from local institutions in Central and South America (see Appendix C)). We used abundance (number of individuals per species) and frequency (number of plots in which a species was present) to quantify the main patterns in taxonomic composition, growth forms, the prevalence of native vs. exotic species, and successional guilds. For successional guilds, we classified species into different groups: non-forest species = species that occur only in open areas or agricultural areas; pioneers = early and late pioneers; intermediate = species from intermediate stages of forest succession; mature forest species = species that occur mainly in mature forests; and secondary species = species for which the only available information indicated that species occurred in secondary forests but there was no indication of whether they were pioneers or intermediate (see Appendix C for details).

#### Assessing species diversity by species richness

The large differences in sample sizes, plot size, and the number of individuals are a challenge for the estimation of species richness using data from different studies^54^. We used the method for rarefaction (interpolation) and extrapolation (prediction) with Hill numbers (^q^D, for species richness, where q = 0), to standardize samples based on sample size^55^ and facilitate the comparison of biodiversity estimates from different studies. To account for differences in overall abundance, sample-based richness estimates were rescaled to the number of individuals sampled using the average number of individuals collected in each sample. Sample-based curves were constructed for each AFS × country combination that had at least 19 plots, as this was the minimum number of plots that allowed us to compare the maximum number of curves (below this there were AFS too few plots to build rarefaction curves). We built rarefaction curves for 18 AFS × country combinations with > 19 plots, while DTP-PAN and LF-BLZ were not included due to their small sample size. All species identified by scientific and vernacular names were included in the analysis. We chose to include data with vernacular names to avoid data loss from some AFS where most individuals were identified with vernacular names (i.e., COCOA-AFS-BLZ). All unidentified species were considered as one class (NO-ID). Since curves were constructed for each AFS × country combination, the chance of inflating richness estimates was low because, within a country, vernacular names are more likely to remain similar. Also, in the case of COCOA-AFS-BLZ, vernacular names corresponded only to one study. All curves were constructed up to a maximum of 250 samples (plots) and 10,000 individuals. Estimates per sample consider community structure while estimates per individual estimate richness assuming a random distribution of all species across the individuals in all the samples collected^54^. Richness within a country was compared at 100 samples and 5,000 individuals for most AFS × country combinations. However, for AFS in Honduras and Belize, comparisons were made at 100 samples and 3,000 individuals to avoid comparing curves extrapolated beyond 2 times the sampling size of the smallest sample within the country which is the maximum limit recommended for species richness by Chao et al*.* (2014). All comparisons of a given practice across countries were made at 100 samples and 3000 individuals. Richness estimates and curves were constructed using the iNEXT library^55,56^ of R 3.1.0 package^57^.

#### Species composition

Species composition was assessed using ordination methods, applying non-metric multidimensional scaling (NMDS) with Euclidean distances to explore the similarities among AFS in terms of their botanical composition. For this analysis, all species identified with vernacular names were pooled together as “unknown” species and were considered separately from ‘NO-ID species (i.e., species that were not identified at all). The number of individuals per species was standardized per area (where density = log(ind + 1)/log(area + 1). In the NMDS, we only obtained the first two axes and for this, we used 1000 randomizations and a tolerance of 0.0001. All analyses were carried out using Qeco^58^.

We also assessed botanical composition in terms of which species were shared across different AFS × country combinations and which ones were unique. For this, we used presence/absence data of a given species in each system and summed the number of total occurrences across all possible AFS × country combinations. If a species was present in all AFS × country combinations, the sum would reach 20. In contrast, a value of 1 would imply that a species only occurred in one of the AFS × country combinations.

Finally, we calculated the average Partial Importance Value Index (IVIp) to assess the relative importance of species across AFS × country combinations. Partial IVI values were estimated by adding the relative density and relative frequencies of species in each of the 20 AFS × country combinations. If a species was not present in a given AFS × country combination, then the IVIp for that AFS × country combination was 0. All species with no ID were considered as one class (NO-ID) and they were included to estimate the relative abundance of species in each plot. The IVIp did not use information about relative dominance since not all plots provided enough information to estimate the basal area of individual plants. Then, the partial IVIs were averaged $\left( {\underline{{IVI_{p} }} } \right)$ across all AFS × country combinations (n AFS × country = 20) including zero values from AFS × country combinations where species were not present. By including the zero values in the average, we avoided potential inflation of $\underline{{IVI_{p} }}$ due to species that occurred in very high densities in only one or two AFS × country combinations. In the results, we report $\underline{{IVI_{p} }}$ and ranges (min and max IVIp).

## Supplementary Information

Supplementary Information.

## Data Availability

Raw data were generated by various individual studies and research projects at CATIE. Compiled and processed data supporting the findings of this study are available from the corresponding author J.C.O. upon request.
