# Supplementary material for: Patterns of shade plant diversity in four agroforestry systems across Central America: a meta-analysis
Source: Sci Rep. 2023 May 26;13:8538. doi: 10.1038/s41598-023-35578-7 (PMC10219940; doi:10.1038/s41598-023-35578-7)
Supplement: Supplementary file 1 — Supplementary Information. [file 41598_2023_35578_MOESM1_ESM.pdf]

Supplementary information:

- Appendix A. Data contributors considered in the study and initial data screening
- Appendix B. Descriptive statistics of plot area, abundance, and plant shade density per agroforestry system for plots included in the working database (n=2,546), including values for 0.05 and 99.5 percentiles
- Appendix C. Growth forms, habitat/successional guilds, and place of origin of tree and shrub species in agroforestry systems in Central America.
- Appendix D. Summary of abundance per family and percentage (and cumulative) of individuals identified at the scientific level.
- Appendix E: Supplementary figure of rarefaction curves by AFS.

Appendix A. Data contributors considered in the study and initial data screening.

A total of 24 sources shared plant inventory or census information (Appendix 1). Sources included individual studies (MSc thesis (n=15) and PhD thesis (n=1)), and complete research projects (n=8) developed between 2003 -2011 by researchers from the Tropical Agricultural Research and Higher Education Center (CATIE). The compilation of original datasets encompassed 3478 plots providing plant inventories of shade species (and in some cases crop species).

In studies part of the PCC project, we found that inventories have been carried out in the same plots at different times, for these studies we only considered one of the inventories while the other replicates were not considered in the analysis. Also, in most of the studies, and particularly for coffee agroforestry systems, we found plots where only 1 shade species was reported. None of these plots (n=908) was included in the analysis. Moreover, we found 24 plots that corresponded to only one agroforestry system in Honduras (Quesungual), that were also excluded, leaving 2456 plots as initial working dataset.

Table A1. Summary of sources that shared shade inventory data including research projects and individual thesis. The table includes the period when field data was collected and number of plots shared in original databases, number of plots that were included in working dataset and reasons for plot exclusion. Numbers in brackets correspond to references related to each data source (listed below).

Sources	Year	Number plots in original databases	Number of plots included in the dataset	Reasons for plot exclusion
Project FRAGMENT [1]	2001-2004	257	221	Live fences or plots with 1 shade species have not been included.
Coffee agroforestry Costa Rica (unpublished data)[2]	2005	40	29	Plots were part of a long-term experiment with different shade combination. Only plots with diverse shade were considered.
Coffee surveys diverse studies (unpublished data) [3,4]	2001-2003	336	319	Plots with only 1 shade species
Project FINNFOR [5,6]	2011-2012	67	37	Plots where area was not reported have not been included
Coffee surveys 3 countries [7,8]	2012	111	38	Most plots had only one shade species (Musa, legumes, or other timber trees), also plots with 2 species, where 1 was not identified were not considered in analysis.
Central America Cocoa Project (PCC)[3]<br>Baseline Project PCC [9]	2007	1234	852	Plots that were covered in the survey of PCC study were not considered. Also plots with only 1 shade species have not been included.
Central America Cocoa Project (PCC)[3]<br>PCC permanent plots [10]	2007	232	216	Plots with only 1 shade species have been excluded.
Central America Cocoa Project (PCC)[3]<br>Project Carbono Biotalamanca [11]	2005	194	32	Most plots from this study have been covered in other studies (PCC and baseline PCC) in different years. Only the plots that have not been included in the other studies were considered for analysis.
15 Thesis CATIE [12-27]	2003-2012	898	740	Plots with only 1 shade species have not been included.<br>1 Thesis that corresponded to Quesungual Agroforestry system was not included. The system was present only in one country.
1 PhD Thesis coffee agroforestry [28]	2008-2011	109	38	Plots with only 1 shade species have not been included.
Total	24	3478	2546	

List of references related to database sources.

1 Harvey, C. *et al.* in *Henry Wallace/CATIE Inter-American Scientific Conference Series. 4. Turrialba (Costa Rica). 1-3 Noviembre 2005* (ed CATIE) 21-26 (CATIE, Turrialba - Costa Rica, 2005).

2 De Melo, E. Evaluación de los sistemas agroforestales con café en fincas vinculadas al consorcio de cooperativas de caficultores de Guanacaste y Montes de Oro- COOCAFE: un aporte a la construcción de la sostenibilidad. . 124 p. (CATIE - FUNCAFOR - COOCAFE - VECO Costa Rica, 2005).

3 Somarriba, E. *et al.* in *Agroforestry and Biodiversity Conservation in Tropical Landscapes: A Synthesis.* (eds G. Schroth *et al.*) 198-226 (Island Press, 2003).

4 Vaast, P. Title: Sustainability of Coffee Agroforestry Systems in Central America; coffee quality and environmental impacts (CASCA) (INCO : International Scientific Cooperation Projects (1998-2002) - Contract number: ICA4-2001-10071 - Third Annual Report, 2004).

5 Detlefsen, G., Marmillod, D., Scheelje Bravo, J. M. & Ibrahim, M. Protocolo para la instalación de parcelas permanentes de medición de la producción maderable en sistemas agroforestales de Centroamérica. (Turrialba, CR, 2012).

6 CATIE & FINNFOR. in *Taller regional sobre investigación a largo plazo en parcelas permanentes de monitoreo en la región mesoamericana: Lecciones y desafíos del presente.* (CATIE).

7 Asigbaase, M. *Biodiversity conservation and carbon sequestration of shade trees in coffee stands in Central America*, University of Grenwich, (2012).

8 Haggar, J., Asigbaase, M., Bonilla, G., Pico, J. & Quilo, A. Tree diversity on sustainably certified and conventional coffee farms in Central America. *Biodivers Conserv* **24**, 1175-1194, doi:10.1007/s10531-014-0851-y (2015).

9 Somarriba, E. *et al.* ¿Cómo diseñamos y ejecutamos el Proyecto Cacao Centroamérica para estimular al sector cacaotero de Centroamérica? *Agroforestería en las Américas*, 111-126 (2013).

10 Orozco, L., Somarriba, E., Villalobos, M., Deheuvels, O. & Andrade, H. (ed Ensenanza Centro Agronomico Tropical de Investigacion y) (Harvard Dataverse, 2014).

11 Andrade, H. J., Segura, M., Somarriba, E. & Villalobos, M. Valoración biofísica y financiera de la fijación de carbono por uso del suelo en fincas cacaoteras indígenas de Talamanca, Costa Rica. *Agroforestería en las Américas* **46**, 45-50 (2008).

12 Hassán Vásquez, J. A. *El ciclo de vida en la producción de leche y la dinámica de las emisiones de gases de efecto invernadero en fincas doble propósito de la península de Azuero, Repbûlica de Panamá* Tesis (Mag. Sc.) thesis, CATIE, (2011).

13 Jiménez Nehring, N. G. *Producción de madera y almacenamiento de carbono en cafetales con cedro (Cedrela odorata) y caoba (Swietenia macrophylla) en Honduras* Tesis (Mag. Sc.) thesis, CATIE, (2012).

14 Leiva Granados, E. R. *Efectos del marco político y legislativo en el aprovechamiento de la madera de sistemas agroforestales del Municipio de El Cuá, Nicaragua* Tesis (Mag. Sc.) thesis, CATIE, (2011).

15 Merlo Caballero, M. E. *Comportamiento productivo del café (Coffea arabica var caturra), el poró (Erythrina poeppigiana), el amarillón (Terminalia amazonia) y el cashá (Chloroleucon eurycyclum) en sistemas agroforestales bajo manejos convencionales y orgánicos en Turrialba, Costa Rica* Tesis (Mag. Sci.) thesis, CATIE, (2007).

16 García Inestroza, E. D. *Evaluación del impacto del uso ganadero sobre suelo y vegetación en el Sistema Agroforestal Quesungual (SAQ) en el sur de Lempira, Honduras* Tesis (Mag. Sci.) thesis, CATIE, (2011).

17 Martínez Salinas, M. A. *Conectividad funcional para aves terrestres dependientes de bosque en un paisaje fragmentado en Matiguás, Nicaragua* Tesis (Mag. Sci.) thesis, CATIE, (2008).

18 Plata Prada, O. F. *Análisis ex ante del aprovechamiento maderable de árboles en potrero, con implementación de prácticas silviculturales, en sistemas silvopastoriles en Esparza, Costa Rica* Tesis (Mag. Sci.) thesis, CATIE, (2012).

19 Pérez Sánchez, E. *Caracterización de sistemas silvopastoriles y su contribución socioeconómica a productores ganaderos de Copán, Honduras* Tesis (Mag. Sci.) thesis, CATIE, (2006).

20 Rosa Cruz, A. *Desafíos de la legislación forestal para el aprovechamiento del recurso maderable en sistemas silvopastoriles del Cayo, Belice* Tesis (Mag. Sci.) thesis, CATIE, (2010).
21 Rosa Cruz, A., Detlefsen, G., Ibrahim, M., de Camino, R. & Galloway, G. Aprovechamiento del recurso maderable en sistemas silvopastoriles de Belice. *Recursos Naturales y Ambiente* **59-60**, 91-98 (2010).
22 Ramírez Sandoval, L. R. *Contribución ecológica y cultural de los sistemas silvopastoriles para la conservación de la biodiversidad en Matiguás, Nicaragua* Tesis (Mag. Sci.) thesis, CATIE, (2007).
23 Ramos Veintimilla, R. A. *Fractioning of soil organic carbon in three types of land uses on cattle farMON in San Miguel de Barranca, Puntarenas-Costa Rica* Tesis (Mag. Sci.) thesis, CATIE, (2003).
24 Scheelje Bravo, J. M. *Incidencia de la legislación sobre el aprovechamiento del recurso maderable en sistemas silvopastoriles de Costa Rica* Tesis (Mag. Sci.) thesis, CATIE, (2009).
25 Scheelje Bravo, J. M., Ibrahim, M. A., Detlefsen, G., Pomareda, C. & Sepúlveda López, C. J. Beneficios financieros del aprovechamiento maderable sostenible en sistemas silvopastoriles de Esparza, Costa Rica. *Agroforestería en las Américas* **48**, 137-145 (2011).
26 Santivañez Galarza, J. L. *Efecto de la estructura, composición y conectividad de las cercas vivas en la comunidad de aves en Río Frío, Costa Rica* Tesis (Mag. Sci.) thesis, CATIE, (2005).
27 Sauceda Olivera, M. *Impacto del arreglo espacial del componente arbóreo en sistemas silvopastoriles sobre el nivel de sombreado y la conectividad estructural de los paisajes en los municipios de Belén y Matiguás, Nicaragua* Tesis (Mag. Sci.) thesis, CATIE, (2010).
28 Cerdán Cabrera, C. R. *Local knowledge regarding trade-offs among coffee productivity and other ecosystem services in a range of different agroforestry systems in Central America* PhD. Thesis thesis, Bangor University (BU) and CATIE, (2012).

Appendix B. Descriptive statistics of plot area, plant shade abundance and density per agroforestry system (AFS) for plots included in the working database (n=2546), including values for 0.05 and 99.5 percentiles.

Agroforestry Practice	Plot area (ha)								
	n	Average	D.E.	Minimum	Maximum	Median	Kurtosis	P(0.05)	P(99.5)
DTP	501	4.36	15.81	0.04	161.0	1.00	63.4	0.04	161.00
COCOA_AF	1126	0.35	0.84	0.07	9.8	0.10	39.2	0.07	6.03
COFFEE_AF	553	0.14	0.13	0.03	0.5	0.10	3.2	0.03	0.50
LF[1]	366	0.51	0.45	0.01	1.6	0.43	-1.2	0.01	1.61
	Abundance (Individuals plot^{-1})								
DTP	501	75.22	139.65	4	1549	35	40.2	4	1096
COCOA_AF	1126	75.07	218.32	1	2725	14	60.2	1	1908
COFFEE_AF	553	35.13	29.40	4	221	26	7.7	4	183
LF	366	42.33	112.00	1	1315	20	66.7	1	957
	Density (Individuals ha^{-1})								
DTP	501	45.17	59.50	0.9	507.0	26.00	20.0	0.9	420
COCOA_AF	1126	172.60	151.62	0.3	2063.9	130.00	26.2	0.3	868
COFFEE_AF	553	340.40	288.97	20.0	2210.0	260.00	8.5	20.0	1830
LF	366	418.79	781.25	1.1	6500.0	66.54	17.9	1.1	5200

DTP = dispersed trees on pastures; COCOA-AFS = cocoa agroforestry; COFFEE-AFS = coffee agroforestry and LF= live fences.
1 For the specific case of live fences which are sampled in lines rather than plots, we assumed a lateral area of 2 m (1m at each side of the fence) to transform fence longitude to plot area, thus if a fence was sampled in a line of 50m the plot area corresponded to 100m2

Appendix C. Growth forms, habitat/successional guilds, and place of origin of tree and shrub species in agroforestry systems in Central America.

All shade species identified with scientific names (genus and species) were characterized by origin (native or exotic), and growth form: tree (T), shrub (SH), small trees (T-SH) palm (PL), climber (CL), large monocots (LM), large herbs (LH). We obtained species information from expert knowledge of the authors (for some of the most well-known species in the region), online databases, (e.g. World Flora Online and GBIF www.gbif.org, last visited in April 2021), peer-reviewed publications and grey literature (e.g. thesis, reports, and other publications from local institutions in Central and South America

Also, for all native woody species (trees and shrubs) identified to species level (n=391), we also collected information on habitat and successional guilds from published sources (peer-reviewed articles, online databases) and grey literature thesis, reports, online publications from universities and local institutions in Central and South America. For a full list of sources see table 3.A and 3.B.

According to the habitat/successional guilds identified in different publications, species were first grouped into 6 classes:

1. Growing in open areas (open areas): species growing in road edges, disturbed sites not associated with agricultural areas, or in general described as "open areas" outside the forest.
2. Growing in farmland (farmland): Species growing within different land uses linked to agriculture or identified in general as agricultural land.
3. Forest Pioneers (pioneer): fast-growing species from earlier stages of succession, heliophytes (short and long-lived), and late pioneers or long-lived pioneers.
4. Forest Intermediate (intermediate): successional stages between pioneer and mature forest.
5. Mature forest (primary): species present or typical of mature forests and shade-tolerant species.
6. Secondary forest (secondary): secondary forest species with no other indication to specific successional guild indicated.

For reporting results regarding habitat/successional classes, the information found in literature was summarized considering all possible combinations for a species, because some species sometimes were reported in more than one habitat/successional class by different sources. The classes considered were:

- Primary: Forest species that were reported as present only in primary forests.
- Intermediate-Primary: forest species reported as intermediate or the combination of intermediate and primary stages of succession.
- Secondary forests: Forest species that were reported as being pioneers, intermediates or present in secondary forests (without indicating any specific stage) or the combination of these classes (does not include non-forest species).
- Forests species with no clear categorization of succession: Forest species that were reported with combinations of different stages of succession from pioneers and mature forests, but only present in forests.
- No forest species: Species that were reported to occur only in open areas, only farmland areas or the combination of these two classes.

- **Generalists:** Species that were reported outside and inside forests in different stages of succession by different sources.

Table C.1 Number of species per successional guild, for all woody (tree and s species:

	NO FOREST	PIONEER	SECONDARY	INTERMEDIATE	PRIMARY	TOTAL
NO FOREST	38	26	11	10	9	94
PIONEER		80	26	17	38	161
SECONDARY			15	5		20
INTERMEDIATE				12	25	37
PRIMARY					70	70
	38	106	52	44	142	382

Table C.2 Summary successional guilds for all native woody species based on richness.

Successional guilds	Number of species	Proportion all species
NO FOREST SPECIES	38	10%
GENERALISTS (FOREST AND NO FOREST SPECIES)	56	15%
SECONDARY FORESTS	143	37%
INTERMEDIATE-PRIMARY	37	10%
PRIMARY	70	18%
FOREST NO CLEAR SUCCESSION	38	10%
TOTAL	382	100%

Table C.3 Number of individuals per successional guild, for all woody species:

	NO FOREST	PIONEER	SECONDARY	INTERMEDIATE	PRIMARY	TOTAL
NO FOREST	18 050	15 102	3 264	24 276	1 075	61 767
PIONEER		15 993	831	1 759	2 377	20 960
SECONDARY			297	32		329
INTERMEDIATE				596	1 276	1 872
PRIMARY					3 489	3 489
	18 050	31 095	4 392	26 663	8 217	88 417

Table C.4 Summary successional guilds for all native woody species based on abundance.

Successional guilds	Number of individuals	Proportion all individuals
NO FOREST SPECIES	18050	20%
GENERALISTS FOREST AND NO FOREST SPECIES	43717	49%
SECONDARY FORESTS	18912	21%
INTERMEDIATE-PRIMARY	1872	2%
PRIMARY	3489	4%
FOREST NO CLEAR SUCCESSION	2377	3%
TOTAL	88417	100%

Table C.5 Number of species per successional guild for singletons (1 individual in the whole dataset).

	NO FOREST	PIONEER	SECONDARY	INTERMEDIATE	PRIMARY	ROW TOTAL
NO FOREST	6	3	1	0	1	11
PIONEER		1	3	2	4	10
SECONDARY			3	1		4
INTERMEDIATE				3	7	10
PRIMARY					15	15
COLUMN TOTAL	6	4	7	6	27	50

Table C.6 Summary successional guilds (%) for singletons (1 individual in the whole dataset).

Successional guilds	Number of species	Proportion all species
NO FOREST	6	12%
GENERALISTS FOREST AND NO FOREST SPECIES	5	10%
SECONDARY FORESTS	10	20%
INTERMEDIATE-PRIMARY	10	20%
PRIMARY	15	30%
FOREST NO CLEAR SUCCESSION	4	8%
	50	100%

Table A3.5. Successional guilds for native woody species identified at the species level. GF= growth form: tree (T), shrub (SH), small trees (T-SH). Successional guilds: O=Open areas; F=Farmland; P=Pioneers; I=Intermediate; MF= mature forest; SF= secondary forest, with no indicationa of a specific status. Species for which no information was found are indicated with grey.

N	Species scientific name	GF	O	F	P	I	MF	SF	Sources
1	*Abarema acreana*	T					1		1
2	*Abarema barbouriana*	T					1		2
3	*Abarema macradenia*	T				1	1		3,4
4	*Abarema idiopoda*	T			1				5
5	*Abarema zollerana*	T							--
6	*Acacia farnesiana*	SH	1						6
7	*Acacia hindsii*	T-SH			1				7
8	*Acacia pennatula*	T-SH						1	8
9	*Acnistus arborescens*	T-SH			1				9
10	*Acosmium panamense*	T				1	1		10
11	*Albizia adinocephala*	T			1			1	2,8
12	*Albizia guachapele*	T			1				2
13	*Albizia niopoides*	T			1				11
14	*Alchornea latifolia*	T-SH			1				12
15	*Alseis yucatanensis*	T			1				13
16	*Alvaradoa amorphoides*	T-SH			1				8
17	*Ampelocera hottlei*	T					1		14
18	*Amphipterygium adstringens*	T-SH			1				15
19	*Amphitecna latifolia*	T-SH		1					2
20	*Anacardium excelsum*	T	1					1	2
21	*Anacardium occidentale*	T			1				16
22	*Andira inermis*	T		1	1		1		2
23	*Annona cherimola*	T		1					6
24	*Annona muricata*	T		1					16
25	*Annona purpurea*	T						1	2
26	*Annona reticulata*	T		1					6
27	*Annona squamosa*	T		1					2
28	*Apeiba membranacea*	T					1		2
29	*Apeiba tibourbou*	T	1	1	1				2
30	*Apoplanesia paniculata*	T-SH			1				17
31	*Aralia excelsa*	T			1	1			10
32	*Ardisia compressa*	T-SH						1	18
33	*Aspidosperma desmanthum*	T					1		2
34	*Aspidosperma excelsum*	T					1		19
35	*Aspidosperma megalocarpon*	T					1		20
36	*Aspidosperma spruceanum*	T					1		21
37	*Astianthus viminalis*	SH				1		1	22
38	*Astronium graveolens*	T		1			1	1	2
39	*Bauhinia divaricata*	T-SH						1	23
40	*Bauhinia ungulata*	T-SH						1	11
41	*Beilschmiedia pendula*	T					1		2

N	Species scientific name	GF	O	F	P	I	MF	SF	Sources
42	*Beilschmiedia riparia*	T							24
43	*Billia rosea*	T				1	1		3,25
44	*Bixa orellana*	T		1					2
45	*Blepharidium guatemalense*	T					1		26
46	*Boehmeria caudata*	SH			1				27
47	*Bourreria huanita*	T			1				28
48	*Brosimum alicastrum*	T			1	1	1		3,16
49	*Brosimum costaricanum*	T				1	1	1	29
50	*Brosimum lactescens*	T				1	1		3,4,30,31
51	*Bucida buceras*	T			1	1			32
52	*Bursera bipinnata*	T			1	1		1	33,34
53	*Bursera simaruba*	T		1	1	1			2,3
54	*Bursera tomentosa*	T-SH			1	1	1		10
55	*Byrsonima crassifolia*	T	1	1					2
56	*Caesalpinia gaumeri*	T			1	1	1		35,36
57	*Caesalpinia violacea*	T-SH			1	1			37
58	*Calatola costaricensis*	T						1	38
59	*Calophyllum brasiliense*	T			1				8
60	*Calycophyllum candidissimum*	T	1	1					2
61	*Capparidastrum frondosum*	T-SH				1	1		39
62	*Carapa guianensis*	T				1	1		3,4
63	*Carica papaya*	T		1					2
64	*Casearia arborea*	T-SH			1			1	2
65	*Casearia corymbosa*	T-SH						1	40
66	*Casearia sylvestris*	T-SH					1	1	2
67	*Casimiroa edulis*	T		1					41
68	*Casimiroa sapota*	T		1					42
69	*Casimiroa tetrameria*	T				1		1	43
70	*Cassia grandis*	T		1	1		1		37,42
71	*Castilla elastica*	T	1	1	1			1	2,16
72	*Castilla tunu*	T					1		44
73	*Cecropia obtusifolia*	T			1				2
74	*Cecropia peltata*	T			1				2
75	*Cedrela odorata*	T	1	1	1	1		1	2
76	*Ceiba aesculifolia*	T	1	1				1	45
77	*Ceiba pentandra*	T	1		1				21
78	*Cespedesia spathulata*	T	1		1				2
79	*Cestrum racemosum*	T-SH					1		46
80	*Chimarrhis parviflora*	T			1	1			4,31
81	*Chloroleucon mangense*	T-SH					1		36
82	*Chomelia spinosa*	T-SH				1	1		10
83	*Chromolaena glaberrima*	SH	1						47
84	*Chrysophyllum cainito*	T			1				48

N	Species scientific name	GF	O	F	P	I	MF	SF	Sources
85	*Chrysophyllum mexicanum*	T-SH					1		49
86	*Chrysophyllum oliviforme*	T				1	1		37
87	*Cinnamomum costaricanum*	T		1			1		50,51
88	*Cinnamomum triplinerve*	T			1			1	2
89	*Clethra lanata*	T	1		1				2
90	*Coccoloba caracasana*	T-SH				1			52,53
91	*Cochlospermum vitifolium*	T			1				16
92	*Cojoba arborea*	T					1		26
93	*Colubrina arborescens*	T			1	1			54
94	*Colubrina spinosa*	T-SH					1		4,55
95	*Cordia alba*	T	1						2
96	*Cordia alliodora*	T	1		1	1			3,8
97	*Cordia bicolor*	T			1	1			3,21
98	*Cordia collococca*	T			1				8
99	*Cordia curassavica*	T-SH				1		1	30
100	*Cordia gerascanthus*	T				1			37
101	*Cordia megalantha*	T					1		56
102	*Cordia panamensis*	T-SH				1	1		10
103	*Cornutia pyramidata*	T-SH			1	1			30,57
104	*Couepia polyandra*	T					1		4
105	*Couratari scottmorii*	T					1		58
106	*Couroupita nicaraguarensis*	T						1	42,59
107	*Crescentia alata*	T-SH			1				16
108	*Crescentia cujete*	SH	1	1					2
109	*Croton draco*	T	1						2
110	*Croton reflexifolius*	T-SH			1			1	54
111	*Cupania belizensis*	T-SH			1		1		30,57
112	*Cupania cinerea*	T-SH	1						2
113	*Cupania dentata*	T	1		1				29
114	*Cupania guatemalensis*	T					1		10
115	*Cupania latifolia*	T	1		1				3,29
116	*Cupressus lusitanica*	T	1	1					6
117	*Curatella americana*	T-SH	1		1			1	2
118	*Dalbergia glomerata*	T			1				8
119	*Dalbergia retusa*	T			1				60
120	*Datura stramonium*	SH	1	1					6
121	*Dendropanax arboreus*	T			1			1	16
122	*Dialium guianense*	T			1				16
123	*Diospyros salicifolia*	T-SH			1	1	1		10
124	*Diospyros johnstoniana*	T-SH					1		61
125	*Diphysa americana*	T	1	1	1				2
126	*Dipteryx oleifera*	T					1		62
127	*Dussia macroprophyllata*	T			1	1	1		3,4,63
128	*Enterolobium cyclocarpum*	T	1	1				1	2,64
129	*Erythrina berteroana*	T	1	1	1				2,37

N	Species scientific name	GF	O	F	P	I	MF	SF	Sources
130	*Erythrina costaricensis*	T	1		1				21
131	*Erythrina fusca*	T	1	1					2
132	*Erythrina hondurensis*	T					1	1	65
133	*Erythrina lanata*	T					1		66
134	*Eugenia guatemalensis*	T-SH						1	67
135	*Eugenia hiraeifolia*	T-SH						1	68
136	*Eugenia hondurensis*	T-SH							--
137	*Eugenia laevis*	T-SH							--
138	*Exostema mexicanum*	T				1	1		10
139	*Ficus americana*	T				1			69
140	*Ficus aurea*	T			1				37
141	*Ficus benjamina*	T	1	1					45
142	*Ficus colubrinae*	T			1			1	30,54
143	*Ficus cotinifolia*	T			1				45
144	*Ficus crassinervia*	T						1	70
145	*Ficus crocata*	T			1	1	1		71
146	*Ficus donnell-smithii*	T			1			1	72
147	*Ficus insipida*	T			1				72
148	*Ficus obtusifolia*	T			1				72
149	*Ficus pertusa*	T			1			1	54
150	*Ficus trigonata*	T					1		73
151	*Ficus citrifolia*	T			1				31
152	*Garcia nutans*	T-SH				1			2
153	*Genipa americana*	T	1	1			1		2
154	*Gliricidia sepium*	T	1	1					74
155	*Godmania aesculifolia*	T-SH					1		31
156	*Guapira costaricana*	T			1	1		1	30,59
157	*Guarea glabra*	T				1	1	1	29
158	*Guarea grandifolia*	T				1	1	1	2,56,75
159	*Guarea rhopalocarpa*	T-SH					1		76
160	*Guazuma ulmifolia*	T	1	1	1				2
161	*Guettarda macrosperma*	T-SH				1	1		10
162	*Gymnanthes lucida*	T-SH					1		77
163	*Gyrocarpus americanus*	T			1				78
164	*Handroanthus chrysanthus*	T			1			1	54
165	*Handroanthus impetiginosus*	T				1			10
166	*Handroanthus guayacan*	T					1		63
167	*Hedyosmum mexicanum*	T			1				79
168	*Heisteria media*	T							80
169	*Heliocarpus appendiculatus*	T			1		1	1	20
170	*Heliocarpus americanus*	T			1				2,81
171	*Hernandia guianensis*	T			1				82
172	*Herrania purpurea*	T-SH					1		2,83,84
173	*Hieronyma alchorneoides*	T	1		1				21
174	*Hirtella guatemalensis*	T-SH					1		85,86

N	Species scientific name	GF	O	F	P	I	MF	SF	Sources
175	*Hirtella racemosa*	T-SH	1		1				87,88
176	*Hura crepitans*	T		1	1	1			2,3
177	*Hymenaea courbaril*	T		1					2
178	*Inga canonegrensis*	T							--
179	*Inga edulis*	T	1	1					2
180	*Inga inicuil*	T			1				18
181	*Inga jinicuil*	T			1				8,18
182	*Inga leiocalycina*	T					1	1	89
183	*Inga micheliana*	T			1				18
184	*Inga nobilis*	T	1		1				2
185	*Inga oerstediana*	T			1				3,8
186	*Inga pavoniana*	T			1				90
187	*Inga punctata*	T			1				8
188	*Inga ruiziana*	T			1				8
189	*Inga vera*	T	1		1			1	2
190	*Jacaratia mexicana*	T					1		54
191	*Jacaratia spinosa*	T			1				3
192	*Jatropha curcas*	T		1					2
193	*Juglans olanchana*	T			1				8
194	*Karwinskia calderonii*	T-SH			1	1			91,92
195	*Koanophyllon pittieri*	T-SH			1				93
196	*Lacmellea panamensis*	T					1		2
197	*Laetia procera*	T			1				4,94
198	*Laetia thamnia*	T					1		21
199	*Laguncularia racemosa*	T-SH			1				35
200	*Leucaena leucocephala*	T-SH			1				95
201	*Leucaena salvadorensis*	T		1					96
202	*Leucaena shannonii*	T-SH			1			1	54
203	*Licania arborea*	T		1	1	1			2,87
204	*Licania platypus*	T				1	1		97
205	*Lippia myriocephala*	T			1				60
206	*Liquidambar styraciflua*	T			1	1			79
207	*Lonchocarpus castilloi*	T			1				8,98
208	*Lonchocarpus costaricensis*	T-SH			1	1	1		10
209	*Lonchocarpus eriocarinalis*	T-SH			1	1			10
210	*Lonchocarpus ferrugineus*	T			1	1	1	1	8,99,100
211	*Lonchocarpus guatemalensis*	T			1				8
212	*Lonchocarpus macrocarpus*	T		1				1	42
213	*Lonchocarpus macrophyllus*	T			1	1		1	31
214	*Lonchocarpus minimiflorus*	T			1				8
215	*Lonchocarpus parviflorus*	T-SH				1			10
216	*Lonchocarpus salvadorensis*	T				1			87,101
217	*Lonchocarpus heptaphyllus*	T-SH	1		1				21
218	*Luehea candida*	T			1				8
219	*Luehea seemannii*	T	1		1				21

N	Species scientific name	GF	O	F	P	I	MF	SF	Sources
220	*Luehea speciosa*	T	1						2
221	*Lysiloma acapulcense*	T-SH			1			1	54
222	*Lysiloma auritum*	T			1	1	1		10
223	*Lysiloma divaricatum*	T				1	1		10
224	*Maclura tinctoria*	T			1				8
225	*Macrohasseltia macroterantha*	T					1	1	58
226	*Magnolia yoroconte*	T				1		1	102
227	*Mammea americana*	T			1				37
228	*Manilkara chicle*	T			1	1	1		10
229	*Manilkara zapota*	T		1			1		5
230	*Marila pluricostata*	T					1		103
231	*Metopium brownei*	T			1				16
232	*Miconia argentea*	T-SH	1	1	1				2
233	*Mimosa tenuiflora*	SH	1	1	1				104
234	*Minquartia guianensis*	T					1		4
235	*Mosquitoxylum jamaicense*	T				1	1	1	30
236	*Muntingia calabura*	T-SH	1	1					2
237	*Myriocarpa longipes*	SH			1			1	30,54
238	*Myrospermum balsamiferum*	T							--
239	*Myrospermum frutescens*	T-SH				1			10
240	*Myroxylon balsamum*	T					1		53
241	*Nectandra lineata*	T			1			1	2
242	*Nectandra martinicensis*	T					1		105
243	*Nectandra membranacea*	T			1	1		1	27
244	*Nectandra turbacensis*	T			1	1		1	106,107
245	*Neea amplifolia*	T-SH							--
246	*Neea laetevirens*	SH					1	1	85,108
247	*Neea psychotrioides*	T-SH			1	1	1	1	30,36
248	*Ochroma pyramidale*	T	1	1	1			1	2
249	*Ocotea austinii*	T					1		109,110
250	*Ocotea helicterifolia*	T-SH					1		111
251	*Ocotea veraguensis*	T				1	1		3,10
252	*Omphalea oleifera*	T				1	1		30
253	*Oreomunnea pterocarpa*	T					1	1	112,113
254	*Oreopanax geminatus*	T					1		54
255	*Ormosia macrocalyx*	T			1		1		2,114
256	*Otoba novogranatensis*	T				1	1		3,4
257	*Pachira quinata*	T			1				60
258	*Parmentiera aculeata*	T-SH					1		35
259	*Peltogyne purpurea*	T				1	1		115
260	*Pentaclethra macroloba*	T				1	1		3,4
261	*Pera arborea*	T					1		31
262	*Persea americana*	T		1					2
263	*Persea schiedeana*	T					1		49
264	*Perymenium grande*	T-SH			1				8

N	Species scientific name	GF	O	F	P	I	MF	SF	Sources
265	*Pimenta dioica*	T			1				60
266	*Pinus caribaea*	T			1			1	16
267	*Pinus oocarpa*	T			1				8
268	*Piper aduncum*	SH			1		1	1	20
269	*Piscidia grandifolia*	T			1				116
270	*Piscidia piscipula*	T					1		49
271	*Pisonia macranthocarpa*	T-SH						1	117
272	*Pithecellobium dulce*	T		1			1		35,118
273	*Pithecellobium lanceolatum*	T-SH			1	1			10
274	*Platymiscium dimorphandrum*	T						1	8
275	*Platymiscium parviflorum*	T		1				1	119
276	*Platymiscium pinnatum*	T					1		21
277	*Platymiscium yucatanum*	T					1		26
278	*Pluchea carolinensis*	SH	1	1					120
279	*Poeppigia procera*	T			1				54
280	*Posoqueria latifolia*	T-SH	1	1	1				2,121
281	*Pouteria amygdalina*	T				1	1		32,122
282	*Pouteria campechiana*	T					1		4
283	*Pouteria fossicola*	T					1		76
284	*Pouteria reticulata*	T					1		3,21
285	*Pouteria sapota*	T					1		26
286	*Prosopis juliflora*	T-SH					1		35
287	*Protium copal*	T-SH					1		26
288	*Protium panamense*	T-SH					1		2
289	*Pseudobombax septenatum*	T	1		1			1	2
290	*Psidium friedrichsthalianum*	T		1					11
291	*Psidium guajava*	T			1				8,41
292	*Psidium salutare*	SH					1		123
293	*Pterocarpus officinalis*	T					1		63
294	*Pterocarpus rohrii*	T					1		21
295	*Quararibea asterolepis*	T				1	1		3
296	*Quassia amara*	T-SH					1		105
297	*Quercus oleoides*	T			1				60
298	*Quercus peduncularis*	T			1			1	54
299	*Rehdera penninervia*	T					1	1	124,125
300	*Rehdera trinervis*	T-SH			1	1	1		10
301	*Rhizophora mangle*	T				1	1		--
302	*Rollinia mucosa*	T			1				126
303	*Rollinia pittieri*	T				1	1		2,3
304	*Roseodendron donnell-smithi*	T			1	1			116
305	*Ruprechtia costata*	T	1	1					2
306	*Sacoglottis trichogyna*	T					1		3,127
307	*Samanea saman*	T	1	1		1	1		10
308	*Sapindus saponaria*	T	1	1					2
309	*Sapium glandulosum*	T	1	1	1				21

N	Species scientific name	GF	O	F	P	I	MF	SF	Sources
310	*Sapium macrocarpum*	T					1		26
311	*Saurauia montana*	T-SH			1		1	1	29
312	*Schefflera morototoni*	T			1				31
313	*Schizolobium parahyba*	T			1				8,16,41
314	*Schoepfia schreberi*	T-SH						1	117
315	*Schoepfia vacciniiflora*	T			1				116
316	*Senegalia polyphylla*	T			1				128
317	*Senna atomaria*	T-SH			1	1			10
318	*Senna hayesiana*	T-SH		1	1			1	121,129
319	*Senna nicaraguensis*	T-SH				1			130
320	*Senna pallida*	SH	1		1			1	23
321	*Senna papillosa*	T-SH			1			1	129,131
322	*Senna reticulata*	T-SH	1						132
323	*Sideroxylon capiri*	T				1			10
324	*Simarouba amara*	T			1				4
325	*Simarouba glauca*	T			1	1	1		37,133,134
326	*Solanum bansii*	T-SH		1					135
327	*Solanum betaceum*	T-SH		1					11
328	*Solanum erianthum*	T-SH	1	1	1				11
329	*Solanum rudepannum*	T-SH			1			1	136
330	*Spondias mombin*	T			1				3,8
331	*Spondias purpurea*	T			1				16
332	*Spondias radlkoferi*	T			1			1	21
333	*Stemmadenia pubescens*	SH					1		137
334	*Sterculia apetala*	T	1	1	1				2,138,139
335	*Stryphnodendron microstachyum*	T			1	1			3,4
336	*Styrax argenteus*	T			1	1			116
337	*Swartzia cubensis*	T					1		10
338	*Swietenia humilis*	T			1				41
339	*Swietenia macrophylla*	T			1	1	1		8,41
340	*Symphonia globulifera*	T-SH					1		21
341	*Tabebuia ochracea*	T		1	1				60
342	*Tabebuia rosea*	T		1	1				2
343	*Tabernaemontana donnell-smithii*	T-SH	1		1			1	29
344	*Tabernaemontana grandiflora*	SH			1	1	1		107,140,141
345	*Tecoma stans*	T			1				16
346	*Terminalia amazonia*	T			1	1	1		5,142
347	*Terminalia lucida*	T					1		11,143
348	*Terminalia oblonga*	T			1				5
349	*Ternstroemia tepezapote*	T-SH			1	1	1		54,111,144
350	*Tetragastris panamensis*	T					1		21
351	*Theobroma angustifolium*	T					1		4,145
352	*Theobroma bicolor*	T		1			1		145,146
353	*Theobroma grandiflorum*	T		1			1		146
354	*Thevetia peruviana*	T-SH		1					11

N	Species scientific name	GF	O	F	P	I	MF	SF	Sources
355	*Thouinidium decandrum*	T				1	1		10
356	*Trema micrantha*	T	1		1				2
357	*Trichilia americana*	T			1				8
358	*Trichilia havanensis*	T					1		30,56
359	*Trichilia hirta*	T			1				2,22
360	*Trichilia martiana*	T			1			1	30,54
361	*Trichilia pallida*	T					1		21
362	*Trichospermum mexicanum*	T			1				94
363	*Triplaris melaenodendron*	T						1	147,148
364	*Trophis mexicana*	T-SH			1		1	1	20
365	*Trophis racemosa*	T				1	1		3,21
366	*Turpinia occidentalis*	T	1		1				21
367	*Ulmus mexicana*	T					1		149
368	*Urera simplex*	SH							--
369	*Vachellia collinsii*	T-SH			1	1			60
370	*Vernonanthura patens*	T-SH			1				150,151
371	*Viburnum hartwegii*	SH				1			144
372	*Virola koschnyi*	T				1	1		3-5
373	*Virola sebifera*	T			1	1	1		2,3
374	*Vismia baccifera*	T-SH	1		1				21
375	*Vitex cooperi*	T			1				5
376	*Vitex gaumeri*	T			1	1	1	1	36
377	*Vochysia guatemalensis*	T	1					1	152
378	*Vochysia ferruginea*	T			1				4
379	*Xylopia aromatica*	T-SH	1	1				1	2
380	*Xylopia sericophylla*	T					1		63
381	*Xylosma flexuosa*	SH				1			136
382	*Xylosma intermedia*	T-SH			1			1	153,154
383	*Zanthoxylum aguilarii*	T					1		54
384	*Zanthoxylum caribaeum*	T				1		1	30,66
385	*Zanthoxylum ekmanii*	T			1	1			3,21
386	*Zanthoxylum elephantiasis*	T			1	1	1		37,155
387	*Zanthoxylum juniperinum*	T					1	1	49,63
388	*Zanthoxylum riedelianum*	T			1				5
389	*Zanthoxylum setulosum*	T			1			1	2,156
390	*Zuelania guidonia*	T		1	1			1	2
391	*Zygia longifolia*	T			1				62

Table A3.6. List of exotic shade species and its place of origin. GF= growth form: tree (T), shrub (SH), small trees (T-SH) palm (PL), climber (CL), large monocots (LM), large herbs (LH).

Species	GF	Region of origin
Acacia mangium	T	Australia
Acacia silvestris	T	Australia
Acrocarpus fraxinifolius	T	Asia
Artocarpus altilis	T	Oceania
Averrhoa carambola	T-SH	Asia
Azadirachta indica	T	India
Caesalpinia coriaria	T-SH	Antillas
Caryodendron orinocense	T	Ven, Col, Ecu
Citrus aurantiifolia	T-SH	SE Asia
Citrus limetta	T-SH	SE Asia
Citrus limon	T-SH	SE Asia
Citrus maxima	T	SE Asia
Citrus reticulata	T	SE Asia
Citrus sp	T-SH	SE Asia
Citrus x paradisi	T	SE Asia
Cocos nucifera	PL	India
Corymbia citriodora	T	Australia
Delonix regia	T	Madagascar
Eriobotrya japonica	T-SH	China
Eucalyptus camaldulensis	T	Australia
Eucalyptus deglupta	T	Australia
Eucalyptus grandis	T	Australia
Eugenia stipitata	T-SH	South America
Ficus carica	T-SH	Asia
Ficus elastica	T	Asia
Gmelina arborea	T	SE Asia
Grevillea robusta	T	Australia
Juglans regia	T	Old World
Khaya senegalensis	T	Africa
Macadamia integrifolia	T	Australia
Mangifera indica	T	Asia
Melia azedarach	T	Indonesia, Australasia
Melicoccus bijugatus	T	South America, Naturalized
Morinda citrifolia	T-SH	Asia
Musa spp.	MUSACEAE	SE Asia
Musa x paradisiaca	MUSACEAE	SE Asia
Myrsine coriacea	T-SH	Brazil
Nephelium lappaceum	T	Indonesia
Persea caerulea	T	Venezuela to Ecuador
Quercus frainetto	T	Europe
Ricinus communis	T-SH	NE Africa
Senna siamea	T	Asia
Skytanthus acutus	SH	Chile, Argentina

Spathodea campanulata	T	Africa
Spondias dulcis	T	Melanesia
Syzygium cumini	T	Asia
Syzygium jambos	T	Asia
Syzygium malaccense	T	Asia
Tamarindus indica	T	Africa
Tectona grandis	T	SE Asia
Vitis vinifera	CL	Mediterranean

Table A3.7. List of species which correspond to non woody growth forms.

Species	Family	Growth form
Acrocomia aculeata	*Arecaceae*	PL
Acrocomia sp	*Arecaceae*	PL
Adenopodia patens	*Fabaceae*	CL
Asterogyne martiana	*Arecaceae*	PL
Astrocaryum mexicanum	*Arecaceae*	PL
Attalea cohune	*Arecaceae*	PL
Bactris gasipaes	*Arecaceae*	PL
Bactris sp	*Arecaceae*	PL
Bambusa bambos	*Poaceae*	LM
Byttneria aculeata	*Malvaceae*	CL
Chamaedorea sp	*Arecaceae*	PL
Chamaedorea tepejilote	*Arecaceae*	PL
Cordyline fruticosa	*Asparagaceae*	LM
Iriartea deltoidea	*Arecaceae*	PL
Raphia taedigera	*Arecaceae*	PL
Sabal mauritiiformis	*Arecaceae*	PL
Urera laciniata	*Urticaceae*	LH
Welfia regia	*Arecaceae*	PL
Yucca gigantea	*Asparagaceae*	LM

List of References

1 Sanchez-Merlos, D. *Criterios ecológicos para la planificación de la conservación en un sector del Corredor Biológico del Atlántico de Nicaragua* MSc. thesis, CATIE, (2006).

2 Condit, R., Perez, R. & Daguerre, N. *Trees of Panama and Costa Rica*. (Princeton University Press, 2011).

3 Castillo Ugalde, M. & Calvo Alvarado, J. C. Monitoreo de la calidad del agua y caracterización de los bosques de la cuenca del Río Carbón. (Instituto Tecnológico de Costa Rica y Corredor Biológico Talamanca-Caribe, https://repositoriotec.tec.ac.cr/bitstream/handle/2238/3101/monitoreo_calidad_rio_carbon.pdf?sequence=2, 2011).

4 Finegan, B., Camacho, M. & Zamora, N. Diameter increment patterns among 106 tree species in a logged and silviculturally treated Costa Rican rain forest. *Forest Ecology and Management* **121**, 159-176, doi:https://doi.org/10.1016/S0378-1127(98)00551-9 (1999).

5 Piotto, D. Growth of native tree species planted in open pasture, young secondary forest and mature forest in humid tropical Costa Rica. *Journal of Tropical Forest Science* **19**, 92-102 (2007).

6 CABI. *Invasive Species Compendium: Datasheets, maps, images, abstracts and full text on invasive species of the world*, <http://www.cabi.org/isc> (2017).

7 Encyclopedia of Life. *EOL v3.*, <https://eol.org/> (2018).

8 Cordero, J. & Boshier, D. H. Vol. 1 (eds Oxford Forestry Institute & CATIE) 1079 (CATIE, Oxford (RU), 2003).

9 Chízmar Fernández, C. *Plantas comestibles de Centroamérica*. (INBio, 2009).

10 Kalacska, M. *et al.* Species composition, similarity and diversity in three successional stages of a seasonally dry tropical forest. *Forest Ecology and Management* **200**, 227-247, doi:https://doi.org/10.1016/j.foreco.2004.07.001 (2004).

11 Fern, K. *The Useful Tropical Plants Database,*, <http://tropical.theferns.info/> (2014).

12 Francis, J. K. Vol. SO-ITF-SM-60 (ed International Institute of Tropical Forestry) (Forest Service USDA, 1993).

13 Slish, D. F., Arvigo, R. & Balick, M. J. Alseis yucatanensis: a natural product from Belize that exhibits multiple mechanisms of vasorelaxation. *Journal of Ethnopharmacology* **92**, 297–302 (2004).

14 González-Di Pierro, A. M. *et al.* Effects of the Physical Environment and Primate Gut Passage on the Early Establishment of Ampelocera hottlei Standley in Rain Forest Fragments. *Biotropica* **43**, 459-466, doi:10.1111/j.1744-7429.2010.00734.x (2011).

15 Guzmán-Pozos, A. M. & Cruz-Cruz, E. Características físicas de frutos de cuachalalate (Amphipterygium adstringens (Schltdl.) Standl) de tres procedencias. *Revista fitotecnia mexicana* **37**, 255-260 (2014).

16 Vázquez-Yanes, C., Batis Muñoz, A. I., Alcocer Silva, M. I., Gual Díaz, M. & Sánchez Dirzo, C. Árboles y arbustos potencialmente valiosos para la restauración ecológica y la reforestación. (CONABIO - Instituto de Ecología, UNAM, 1999).

17 Rincón, E. & Huante, P. Growth responses of tropical deciduous tree seedlings to contrasting light conditions. *Trees* **7**, 202-207, doi:10.1007/BF00202074 (1993).

18 Instituto Nacional de Biodiversidad (INBio). *Atlas de la Biodiversidad de Costa Rica (CRBio)* <http://www.crbio.cr> (2020).

19 Missouri Botanical Garden. *Tropicos.org - Flora Mesoamericana*, <http://legacy.tropicos.org/Name/1800114?projectid=3> (14 Sep 2020).

20 Levy Tacher, S. I., Aguirre Rivera, J. R., Martínez Romero, M. M. & Durán Fernández, A. Caracterización del uso tradicional de la flora espontánea en la comunidad lacandona de lacanhá, chiapas, méxico. *Interciencia* **27**, 512-520 (2002).

21 Comita, L. S., Aguilar, S., Pérez, R., Lao, S. & Hubbell, S. P. Patterns of woody plant species abundance and diversity in the seedling layer of a tropical forest. *Journal of Vegetation Science* **18**, 163-174, doi:10.1658/1100-9233(2007)18[163:powpsa]2.0.co;2 (2007).
22 Martínez-Garza, C., Osorio-Beristain, M., Valenzuela-Galván, D. & Nicolás-Medina, A. Intra and inter-annual variation in seed rain in a secondary dry tropical forest excluded from chronic disturbance. *Forest Ecology and Management* **262**, 2207-2218, doi:https://doi.org/10.1016/j.foreco.2011.08.013 (2011).
23 Rojas Chávez, S. & Vibrans, H. *Malezas de México*, <http://www.conabio.gob.mx/malezasdemexico/caesalpinaceae/bauhinia-divaricata/fichas/ficha.htm> (2009).
24 Nishida, S. *Revisional study of neotropical Beilschmiedia species (Lauraceae) with special reference to leaf anatomy*, Kyoto University, (1998).
25 González, R. T. & Montoya, G. E. 32 p. (2014).
26 Ochoa-Gaona, S., Hernández-Vázquez, F., De Jong, B. H. J. & Gurri-García, F. D. Pérdida de diversidad florística ante un gradiente de intensificación del sistema agrícola de roza-tumba-quema: Un estudio de caso en la selva lacandona, Chiapas, México. *Boletín de la Sociedad Botánica de México* **81**, 65-80 (2007).
27 Siminski, A., Fantini, A. C., Guries, R. P., Ruschel, A. R. & dos Reis, M. S. Secondary Forest Succession in the Mata Atlantica, Brazil: Floristic and Phytosociological Trends. *ISRN Ecology* (2011).
28 Arellano-Rodríguez, J. A., Flores, J. S., Tun, J. & Cruz-Bojórquez, M. M. Nomenclatura, forma de vida, uso, manejo y distribución de las especies vegetales de la Península de Yucatán. *Etnoflora Yucatanense* **20**, 1-815 (2003).
29 Benitez, M., Martinez-Ramos, M. & Ceccon, E. in *Life Forms and Dynamics in Tropical Forests* Vol. Dissertationes Botanicae, Band 346 *Dissertationes Botanicae, Band 346* (eds G. Gottsberger, S. Liede, & R. Lücking) 185-203 (Gebrueder Borntraeger Berlin, 2001).
30 Arroyo-Rodriguez, V., Dunn, J., Ben'itez-malvido, J. & Mandujano, S. Angiosperms , Los Tuxtlas Biosphere Reserve , Veracruz , Mexico. *Check List* **5**, 787-799 (2009).
31 Vargas Fonseca, L. *Análisis de una cronosecuencia de bosques tropicales del corredor biológico Osa, Costa Rica*, Instituto Tecnológico de Costa Rica, (2011).
32 Galleti, H. A. Documento Técnico Unificado De Aprovechamiento Forestal En La Sociedad El Achiotal S.P.R. De R.I., Municipio De Bacalar, Quintana Roo. (Documento Técnico Unificado De Aprovechamiento Forestal en la sociedad EL Achotal SPR de RI, 2015).
33 Laurenceau, M. & Soto-Pinto, L. Sistemas agroforestales para la adaptación al cambio climático en el área protegida La Frailescana, Chiapas, México. *Sociedades Rurales, Produccion y Medio Ambiente* **15**, 19-49 (2015).
34 Newton, A. C. *et al.* Forest Landscape Restoration in the Drylands of Latin America. *Ecology and Society* **17**, doi:10.5751/ES-04572-170121 (2012).
35 Rico-Gray, V., Palacios Ríos, M., Lira, R. & Martínez, J. La Interacción estabilidad-sucesión, un ejemplo: la vegetación costera del estado de Yucatán, México. *Brenesia* **28**, 1-11 (1987).
36 Sanaphre-Villanueva, L. *et al.* Patterns of plant functional variation and specialization along secondary succession and topography in a tropical dry forest. *Environmental Research Letters* **12**, 055004, doi:10.1088/1748-9326/aa6baa (2017).
37 Herrera-Peraza, R. A. *et al.* A new hypothesis on humid and dry tropical forests succession. *Acta Botánica Cubana* **215**, 232-280 (2016).
38 Instituto de Ciencias Naturales, Facultad de Ciencias & Universidad Nacional de Colombia. *Colecciones en Línea*, < http://www.biovirtual.unal.edu.co> (2004).
39 Castellanos Castro, C. *Functional analysis of secondary tropical dry forests in a region of the Colombian caribbean*, Bournemouth University, (2013).
40 Janzen, D. H. in *Ecosystem Management - Selected Readings* (eds Fred B. Samson & Fritz L. Knopf) 192-202 (Springer-Verlag New York, 1996).

41 Orwa, C., Mutua, A., Kindt, R., Jamnadass, R. & Simons, A. *Agroforestree Database: a tree reference and selection guide version 4.0.*, <http://apps.worldagroforestry.org/treedb2/index.php> (2009).
42 Sánchez Merlos, D. *et al.* Vegetation diversity, composition and structure in a cattle agro-landscape of Matiguás, Nicaragua. *Revista de Biología Tropical* **53**, 387-414 (2005).
43 Carreón-Santos, R. J. & Valdez-Hernández, J. I. Tree structure and diversity of secondary vegetation derived from a semi-evergreen tropical forest in Quintana Roo. *Revista Chapingo Serie Ciencias Forestales y del Ambiente* **20**, 119-130 (2014).
44 Herwitz, S. R. *Regeneration of Selected Tropical Tree Species in Corcovado National Park, Costa Rica*. (University of California Press, 1981).
45 Gargiullo, M., Magnuson, B. & Kimball, L. *A Field Guide to the Plants of Costa Rica*. 512 p. (Oxford University Press, 2008).
46 Guariguata, M. R. & Finegan, B. (CATIE).
47 Escobar-Ocampo, M. C. & Ochoa-Gaona, S. Estructura y composición florística de la vegetación del Parque Educativo Laguna Bélgica, Chiapas, México. *Revista mexicana de biodiversidad* **78**, 391-419 (2007).
48 Parker, I. M. *et al.* Domestication Syndrome in Caimito (Chrysophyllum cainito L.): Fruit and Seed Characteristics. *Economic Botany* **64**, 161-175, doi:10.1007/s12231-010-9121-4 (2010).
49 López-Gómez, A. M., Williams-Linera, G. & Manson, R. H. Tree species diversity and vegetation structure in shade coffee farms in Veracruz, Mexico. *Agriculture, Ecosystems & Environment* **124**, 160-172, doi:https://doi.org/10.1016/j.agee.2007.09.008 (2008).
50 Monro, A. K. *et al.* A first checklist to the vascular plants of La Amistad International Park (PILA), Costa Rica-Panama. *Phytotaxa* **322**, 1-283 (2017).
51 Zúñiga, C., Somarriba, E. & Sánchez, V. Tipologías cafetaleras de la Reserva Natural Miraflor - Moropotente, Estelí, Nicaragua. *Agroforestería en las Américas* **41-42**, 105-111 (2004).
52 Garzón, N. V., Córdoba, M. P. & Gutiérrez, J. C. Construcción participativa de estrategias de restauración ecológica en humedales del Magdalena Medio, Colombia: una herramienta para el ordenamiento ambiental territorial. *Biota Colombiana* **15**, 58-86 (2014).
53 Proyecto Ecológico Azuero. *Base de Datos de Plantas*, <http://proecoazuero.org/base-de-datos-de-plantas/> (w.y.).
54 Rocha-Loredo, A. G., Ramírez-Marcial, N. & González-Espinosa, M. Riqueza y diversidad de árboles del bosque tropical caducifolio en la depresión central de chiapas. *Boletín de la Sociedad Botánica de México*, 89-103 (2010).
55 Lieberman, D., Lieberman, M., Hartshorn, G. & Peralta, R. Growth Rates and Age-Size Relationships of Tropical Wet Forest Trees in Costa Rica. *Journal of Tropical Ecology* **1**, 97-109 (1985).
56 Martínez-Garza, C., Peña, V., Ricker, M., Campos, A. & Howe, H. F. Restoring tropical biodiversity: Leaf traits predict growth and survival of late-successional trees in early-successional environments. *Forest Ecology and Management* **217**, 365-379, doi:https://doi.org/10.1016/j.foreco.2005.07.001 (2005).
57 Ferguson, B. G., Vandermeer, J., Morales, H. & Griffith, D. M. Post-Agricultural Succession in El Petén, Guatemala. *Conservation Biology* **17**, 818-828 (2003).
58 Quesada Monge, R., Castillo-Ugalde, M., Lobo Segura, J. & Barrantes, G. Demografía de especies maderables de la Península de Osa. (Instituto Tecnológico de Costa Rica, Costa Rica, 2010).
59 Taylor, B. W. An Outline of the Vegetation of Nicaragua. *Journal of Ecology* **51**, 27-54, doi:10.2307/2257504 (1963).
60 Hilje, B., Calvo-Alvarado, J., Jiménez-Rodríguez, C. & Sánchez-Azofeifa, A. Tree Species Composition, Breeding Systems, and Pollination and Dispersal Syndromes in Three Forest Successional Stages in a Tropical Dry Forest in Mesoamerica. *Tropical Conservation Science* **8**, 76-94, doi:10.1177/194008291500800109 (2015).

61 Wallnöfer, B. A revision of neotropical Diospyros (Ebenaceae): part 2. *Annalen des Naturhistorischen Museums in Wien. Serie B für Botanik und Zoologie* **110**, 173-211 (2008).
62 Vargas G, G. & Cordero S, R. A. Photosynthetic responses to temperature of two tropical rainforest tree species from Costa Rica. *Trees* **27**, 1261-1270, doi:10.1007/s00468-013-0874-0 (2013).
63 Vandermeer, J., Zamora, N., Yih, K. & Boucher, D. Regeneración inicial en una selva tropical en la costa caribeña de Nicaragua después del huracán Juana. *Revista de Biología Tropical* **38**, 349-357 (1990).
64 Gonzales, E., Hamrick, J. L., Smouse, P. E., Trapnell, D. W. & Peakall, R. The Impact of Landscape Disturbance on Spatial Genetic Structure in the Guanacaste Tree, Enterolobium cyclocarpum (Fabaceae). *Journal of Heredity* **101**, 133-143, doi:10.1093/jhered/esp101 (2010).
65 Doblado Amador, L. S. *Identificación y caracterización de tipos de bosque y su relación con variables ambientales, en un paisaje fragmentado al Norte de Honduras*, Centro Agronómico Tropical de Investigación Y Enseñanza, (2011).
66 Alvarez-Añorve, M. Y., Quesada, M., Sánchez-Azofeifa, G. A., Avila-Cabadilla, L. D. & Gamon, J. A. Functional regeneration and spectral reflectance of trees during succession in a highly diverse tropical dry forest ecosystem. *American Journal of Botany* **99**, 816-826 (2012).
67 Harvey, C. A. & Haber, W. A. Remnant trees and the conservation of biodiversity in Costa Rican pastures. *Agroforestry Systems* **44**, 37-68, doi:10.1023/a:1006122211692 (1998).
68 Granda Moser, V., Finegan, B., Ramos Bendana, Z., Detlefsen, G. & Molina, A. Potencial de manejo de bosques restaurados por sucesión natural secundaria en Guanacaste, Costa Rica: Composición, diversidad y especies maderables. (Turrialba, Costa Rica, 2015).
69 López Montenegro, J. & Rusconi Luna, W. C. *Análisis de la sucesión primaria en las lavas de Quezaltepeque, La Libertad*, Universidad de el Salvador, (2005).
70 Jiménez Lang, N. *Heterogeneidad del paisaje y la diversidad arbórea y de aves en ambientes humanizados de la Planicie Costera del Pacífico, Chiapas, México*, El Colegio de la Frontera Sur (2017).
71 Granados-Victorino, R. L., Sánchez-González, A., Martínez-Cabrera, D. & Octavio-Aguilar, P. Estructura y composición arbórea de tres estadios sucesionales de selva mediana subperennifolia del municipio de Huautla, Hidalgo, México. *Revista mexicana de biodiversidad* **88**, 122-135 (2017).
72 Barreno Freire, S. N. & Pulupa Salguero, G. P. *Composición y estructura de la Herpetofauna en dos tipos de bosque en la parroquia de Shushufindi, provincia de Sucumbíos, período 2011 - 2012*, UCE, (2012).
73 Figueroa, J. C., Totti, L., Lugo, A. E. & Woodbury, R. O. Structure and composition of moist coastal forest in Dorado, Puerto Rico. (Forest Service Southern Forest Experiment Station, New Orleans, Louisiana, 1984).
74 Glander, K. & Nisbett, R. Community structure and species density in tropical forest associations in Guanacaste Province, Costa Rica. *Brenesia* **45-46**, 113-142 (1996).
75 López-Pérez, D., Castillo-Acosta, O., Zavala-Cruz, J. & Hernández-Trejo, H. Estructura y composición florística de la vegetación secundaria en tres regiones de la Sierra Norte de Chiapas, México. *Polibotánica*, 1-23 (2014).
76 Murray, K. G. *et al.* in *Post-Agricultural Succession in the Neotropics* (ed Randall W. Myster) 192-215 (Springer, 2008).
77 Carrington, M. E., Ross, M. S. & Basit, A. F. Posthurricane Seedling Structure in a Multi-aged Tropical Dry Forest: Implications for Community Succession. *Biotropica* **47**, 536-541, doi:10.1111/btp.12244 (2015).
78 Uasuf, A., Tigabu, M. & Oden, P. C. Soil seed banks and regeneration of neotropical dry deciduous and gallery forests in Nicaragua. *Bois et forets des tropiques* **299**, 49-62 (2009).

79 Muñiz-Castro, M. A., Williams-Linera, G. & Martinez-Ramos, M. *Dispersal mode, shade tolerance, and phytogeographical affinity of tree species during secondary succession in tropical montane cloud forest*. Vol. 213 (2012).
80 Gomez-Dominguez, H., Perez-Farrera, M. A., Espinoza-Jimenez, J. A. & Marquez-Reynoso, M. I. Listado florístico del parque nacional Palenque, Chiapas, México. *Botanical Sciences* **93**, 559-578 (2015).
81 Aguirre, N., Palomeque, X., Weber, M., Stimm, B. & Gunter, S. in *Silviculture in the Tropics* (eds S. Gunter, M. Weber, Bernd Stimm, & R. Mosandl) 560 p. (Springer-Verlag Berlin Heidelberg, 2011).
82 Gonzalez, V. & Delgado, J. E. The vegetation associated to the set of recent and subrecent islands of the Río Grande del Orinoco estuary. Sector Merejina, Delta Amacuro state, Venezuela. *Biollania Edic. Esp.* **15**, 561-582 (2017).
83 Uriarte, M., Condit, R., Canham, C. D. & Hubbell, S. P. A spatially explicit model of sapling growth in a tropical forest: does the identity of neighbours matter? *Journal of Ecology* **92**, 348-360, doi:doi:10.1111/j.0022-0477.2004.00867.x (2004).
84 Álvarez Salas, L. M., Gálvez Abadía, A. & Salazar Zapata, J. C. Etnobotánica del Darién Caribe colombiano: los frutos del bosque. *Etnográfica: Revista do Centro em Rede de Investigação em Antropologia* **20**, 163-193 (2016).
85 Araya, J. A. *Efectos del huracán Otto sobre la estructura y composición florística de un bosque primario en Boca Tapada de Pital, San Carlos, Costa Rica*, Instituto Tecnológico De Costa Rica, (2017).
86 Pineda Barría, J. A. *Alternativas de manejo forestal para bosques primarios muy intervenidos: Estudio de caso en Finca Elia María, Los Chiles, Costa Rica*, Centro Agronómico Tropical de Investigación Y Enseñanza, (2012).
87 Leiva, J. A., Rocha, O. J., Mata, R. & Gutiérrez-Soto, M. V. Cronología de la regeneración del bosque tropical seco en Santa Rosa, Guanacaste, Costa Rica. II. La vegetación en relación con el suelo. *International JOurnal of Tropical Biology* **57**, 817-836 (2009).
88 Peña-Claros, M. Changes in Forest Structure and Species Composition during Secondary Forest Succession in the Bolivian Amazon. *BIOTROPICA* **35**, 450-461, doi:10.1646/01078 (2003).
89 Eguiguren, P. A. *Los efectos de intervenciones forestales y la variabilidad climática sobre la dinámica a largo plazo de bosques tropicales en el noreste de Costa Rica* MSc. thesis, Centro Agronómico Tropical De Investigación Y Enseñanza, (2013).
90 Pacheco Gómez, T., Burga Alvarado, R., Angulo Ruiz, P. A. & Torres Vásquez, J. in *Geoecología y desarrollo Amazónico: estudio integrado en la zona de Iquitos* (eds R. Kalliola & S. Flores Paitán) 389-416 (Annales Universitatis Turkuensis Ser A11 Tomo 114, 1998).
91 Castro-Marín, G., Tigabu, M., González-Rivas, B. & Oden, P. C. A chronosequence analysis of forest recovery on abandoned agricultural fields in Nicaragua. *Journal of Forestry Research* **20**, 213-222 (2009).
92 Medina-Amaya, M. J. *Análisis del estado de sucesión secundaria de la zona boscosa comprendida en el municipio de Cinquera, departamento de Cabañas, El Salvador.*, Universidad de El Salvador, (2003).
93 De la Peña-Domene, M. & Martínez-Garza, C. Integrating Density into Dispersal and Establishment Limitation Equations in Tropical Forests. *Forests* **9**, 570 (2018).
94 Gomide, G. L. A., Finegan, B., Sanquetta, C. R. & Silva, J. N. M. in *Manejo integrado de florestas umidas neotropicais por industrias e comunidades: aplicando resultados de pesquisa, envolvendo atores e definindo politicas publicas: Simposio Internacional da IUFRO.* (eds C. Sabogal & J.N. Macedo-Silva) 124-139 (CIFOR and Embrapa Amazonia Oriental).
95 Centro Agronómico Tropical de Investigación y Enseñanza. Vol. Serie Técnica - Informe técnico No 166 (Costa Rica, 1991).

96 Boshier, D. in *Forest Genetic Resources Training Guide* (eds D. Boshier, M. Bozzano, J. Loo, & P. Rudebjer) (Bioversity International, 2011).
97 Levy-Tacher, S. I. & Rivera, J. R. A. Successional Pathways Derived from Different Vegetation Use Patterns by Lacandon Mayan Indians. *Journal of Sustainable Agriculture* **26**, 49-82, doi:10.1300/J064v26n01_06 (2005).
98 Pennington, T. & Sarukhán, J. *Árboles tropicales de México : manual para la identificación de las principales especies*. 3era edición edn, 523 p. (UNAM, 2005).
99 Ortiz Malavasi, E., Chazdon, R. L. & Vílchez Alvarado, B. Línea base de estudio de la biodiversidad, servicios ambientales y valores para la conservación de bosques secundarios y maduros en el Corredor Biológico Osa. 117 p. (Instituto Tecnológico De Costa Rica - Escuela de Ingeniería Forestal, Costa Rica, 2011).
100 Morales-Salazar, M. *Composición florística, estructura, muestreo diagnóstico y estado de conservación de una cronosecuencia de bosques tropicales del corredor biológico Osa, Costa Rica*, Instituto Tecnológico De Costa Rica, (2010).
101 Sánchez Rodríguez, E. V., López Mata, L., García Moya, E. & Cuevas Guzmán, R. Estructura, composición florística y diversidad de especies leñosas de un bosque mesófilo de montaña en la sierra de Manantlán, Jalisco. *Boletín de la Sociedad Botánica de México* **73**, 17-34 (2003).
102 Finegan, B., Palacios, W., Zamora, N. & Delgado, D. in *Criteria and Indicators for sustainable forest management* (eds J. Raison, A. Brown, & D. Flinn) 341-378 (CABI, 2001).
103 Letcher, S. G. *et al.* Environmental gradients and the evolution of successional habitat specialization: a test case with 14 Neotropical forest sites. *Journal of Ecology* **103**, 1276-1290, doi:doi:10.1111/1365-2745.12435 (2015).
104 Somarriba, E. Regeneración natural de maderables en campos agrícolas. *Agroforestería en las Américas* **6**, 31-34 (1999).
105 Oatham, M. P. & Ramnarine, S. Dynamics of pioneer and primary successional stage trees in a logged Trinidadian tropical rainforest and the influence of drought *Tropical Ecology* **47**, 13-26 (2006).
106 Adame, P., Brandeis, T. J. & Uriarte, M. Diameter growth performance of tree functional groups in Puerto Rican secondary tropical forests. *2014* **23**, 12, doi:10.5424/fs/2014231-03644 (2014).
107 Pulido R., E. N. *Diversidad funcional de especies arbóreas en dos estados contrastantes de sucesion en un bosque seco tropical en Cundinamarca*, Pontificia Universidad Javeriana, (2018).
108 Sánchez Merlos, D., Finegan, B., Harvey, C. A. & Delgado, D. Tipos de bosques en el sector sur del Corredor Biológico del Atlántico, Nicaragua. *Recursos Naturales y Ambiente*, 48-56 (2006).
109 DeVries, S. & Warners, D. Nine year assessment of soil treatment on aguacatillo (Nectandra cufodontisii and Ocotea austinii, Lauraceae) seedling success in the restoration of a Costa Rican cloud forest. *Brenesia* **81-82**, 29-36 (2014).
110 Instituto Costaricense Electricidad. Estudio impacto ambiental proyecto línea de transmisión Tarbaca – anillo sur 230 kv. 500 p. (Costa Rica, 2007).
111 del Castillo, R. F. & Ríos, M. A. P. Changes in seed rain during secondary succession in a tropical montane cloud forest region in Oaxaca, Mexico. *Journal of Tropical Ecology* **24**, 433-444, doi:10.1017/S0266467408005142 (2008).
112 Veintimilla, D. A. *Identificación y caracterización de tipos de bosque tropical sobre un gradiente altitudinal en Costa Rica: el caso "Caribe-Villa Mills"* MSc. thesis, CATIE, (2013).
113 Ortega, M. *Ecología del paisaje y caracterización de la cobertura forestal, según piso altitudinal de la microcuenca del río La Balsa, Costa Rica, para establecer criterios para el manejo integrado de cuencas*, Instituto Tecnológico De Costa Rica, (2012).

114 Pérez-Hernández, I., Ochoa-Gaona, S., Vargas-Simón, G., Mendoza-Carranza, M. & González-Valdivia, N. A. Germinación y supervivencia de seis especies nativas de un bosque tropical de Tabasco, México. *Madera y bosques* **17**, 71-91 (2011).
115 Morales-Salazar, M. S., Vílchez-Alvarado, B., Chazdon, R. L., Ortiz-Malavasi, E. & M., G.-B. Estructura, composición y diversidad vegetal en bosques tropicales del Corredor Biológico Osa, Costa Rica. *Revista Forestal Mesoamericana Kurú (Costa Rica)* **10**, 2215-2504 (2013).
116 Evans-Cabrera, M. A. *Caracterización de la vegetación natural de sucesión primaria en el Parque Nacional Volcán Pacaya y Laguna de Calderas, Guatemala* MSc. thesis, CATIE, (2006).
117 Derroire, G. *Secondary Succession in Tropical Dry Forests: Drivers and Mechanisms of Forest Regeneration* PhD. Thesis thesis, Bangor University, SLU, (2016).
118 Purdue University. *NewCROP (New Crops Resource Online Program)*, 2017).
119 Esquivel, J. M. *Regeneracion natural de arboles y arbustos en potreros activos en Muy Muy, Matagalpa, Nicaragua* MSc. thesis, CATIE, (2005).
120 Fundora-Mayor, Z. *et al.* in *Seed systems and crop genetic diversity on-farm: Proceedings of a Workshop, 16–20 September 2003, Pucallpa, Peru* (eds Devra I. Jarvis, Ricardo Sevilla-Panizo, José Luis Chávez-Servia, & Toby Hodgkin) 68-77 (IPGRI, 2003).
121 Sanchez-Clavijo, L. M., Velez, J. G., Duran, S. M., GArcia, R. & Botero, J. E. Estudio regional de la biodiversidad en los paisajes cafeteros de Tamesis, Antioquia. (CENICAFE, Colombia, 2010).
122 Martínez, E. & Galindo-Leal, C. La vegetación de Calakmul, Campeche, México: Clasificación, descripción y distribución. *Boletín de la Sociedad Botánica de México* **71**, 7-32 (2002).
123 Córdoba-Córdoba, S., Borja Acosta, K. G. & Medina Uribe, C. A. Caracterización Rápida de Flora y Fauna Gachantivá Boyacá. Reservas Naturales de la Sociedad Civil Cochahuaira, Furatena, Buenavista. (Instituto de Investigación de Recursos Biológicos Alexander von Humboldt, Colombia, 2017).
124 Manzanero, M. & Pinelo, G. (WWF Centroamérica, 2004).
125 Gonzalez-Valdivia, N. *et al.* Análisis comparativo de la estructura, diversidad y composición de comunidades arbóreas de un paisaje agropecuario en Tabasco, México. *Revista Mexicana de Biodiversidad* **83**, 83-89 (2012).
126 Geilfus, F. *El árbol al servicio del agricultor: Manual de agroforesteria para el desarrollo rural - Volumen 2 guía de especies*. (ENDA-CARIBE y CATIE, 1994).
127 Hammel, B. E., Grayum, M. H., Herrera, C. & Zamora, Z. in *(Monographs in Systematic Botany from the Missouri Botanical)* 993 p. (2007).
128 Ferreira Nunes, Y. *et al.* in *Tropical Dry Forests in the Americas: Ecology, Conservation, and Management* (eds A. Sanchez-Azofeifa, J.S. Powers, G.W. Fernandes, & M. Quesada) 325-349 (CRC Press, 2013).
129 Murakami, S. *Contribucion de los pagos por servicios ambientales (PSA) en la restauracion de bosques secundarios en Hojancha, Costa Rica* MSc. thesis, CATIE, (2008).
130 Barahona, T. *The impact of human practices on forest remnants: People and conservation in a small nature reserve in Western Nicaragua* MSc. thesis, Ohio University, (2001).
131 Zahawi, R. A. *et al.* The Effect of Restoration Treatment Soils and Parent Tree on Tropical Forest Tree Seedling Growth. *Open Journal of Forestry* **5**, 154-161 (2015).
132 Parolin, P. Senna reticulata (Willd.) H. S. Irwin & Barneby (Fabaceae) as "Pasture Killer" ("Matapasto") pioneer tree in amazonian floodplains. *Ecología Aplicada* **4**, 41-46 (2005).
133 Ross, M. S., Carrlngton, M., Flynn, L. J. & Ruiz, P. L. Forest Succession in Tropical Hardwood Hammocks of the Florida Keys: Effects of Direct Mortality from Hurricane Andrew1. *Biotropica* **33**, 23-33, doi:doi:10.1111/j.1744-7429.2001.tb00154.x (2001).
134 Navarro, M. A. & Granados, D. Dinamica sucesional en un bosque tropical afectado por disturbios en la zona norte de Quintana Roo. *Revista Chapingo Ciencias Forestales* **1**, 5-16 (1997).

135 Galloway, G. & Beer, J. Oportunidades para fomentar la silvicultura en cafetales en América Central. (CATIE, Turrialba - Costa Rica, 1997).
136 Juárez-Fragoso, M. A., López-Acosta, J. C. & Velázquez-Rosas, N. Contribución al conocimiento ecológico y florístico de un palmar dominado por *Sabal mexicana* mart. al sur del estado de Veracruz, México. *Polibotánica* **44**, 51-66 (2017).
137 Williams-Linera, G., Alvarez-Aquino, C., Hernández-Ascención, E. & Toledo, M. Early successional sites and the recovery of vegetation structure and tree species of the tropical dry forest in Veracruz, Mexico. *New Forests* **42**, 131-148, doi:10.1007/s11056-010-9242-8 (2011).
138 Janzen, D. H. Escape in Space by Sterculia Apetala Seeds from the Bug Dysdercus Fasciatus in a Costa Rican Deciduous Forest. *Ecology* **53**, 350-361, doi:doi:10.2307/1934092 (1972).
139 Kennard, D. K. Secondary forest succession in a tropical dry forest: patterns of development across a 50-year chronosequence in lowland Bolivia. *Journal of Tropical Ecology* **18**, 53-66, doi:10.1017/S0266467402002031 (2002).
140 Olascuaga Vargas, D., Mercado Gomez, J. & L.R., S. M. Análisis de la vegetación sucesional en un fragmento de bosque seco tropical en Toluviejo-Sucre (Colombia). *Colombia Forestal* **19**, 23-40 (2016).
141 Castellanos-Castro, C. & Newton, A. C. Environmental Heterogeneity Influences Successional Trajectories in Colombian Seasonally Dry Tropical Forests. *Biotropica* **47**, 660-671, doi:10.1111/btp.12245 (2015).
142 Vandermeer, J. & Granzow de la Cerda, I. Height dynamics of the thinning canopy of a tropical rain forest: 14 years of succession in a post-hurricane forest in Nicaragua. *Forest Ecology and Management* **199**, 125-135, doi:https://doi.org/10.1016/j.foreco.2004.05.033 (2004).
143 CONIF-PIZANO. Manejo y Conservacion del ecosistema Catival. 118 p. (Colombia, 1999).
144 Valencia, V., Naeem, S., García-Barrios, L., West, P. & Sterling, E. J. Conservation of tree species of late succession and conservation concern in coffee agroforestry systems. *Agriculture, Ecosystems & Environment* **219**, 32-41, doi:https://doi.org/10.1016/j.agee.2015.12.004 (2016).
145 Castañeda-Langlois, H. *Ethnobotanical analysis of different successional stages as sources of wild edible plants for the Guaymi people in Costa Rica* MSc. thesis, University of FLorida, (2004).
146 Goetsch, E. *Natural succession of species in agroforestry and in soil recovery* (http://www.agrofloresta.net/artigos/agroforestry_1992_gotsch.pdf, 1992).
147 Rojas-Chaves, P. A., Vilchez-Alvarado, B., Moya-Roque, R. & Sasa-Marin, M. Combustibles forestales superficiales y riesgo de incendio en dos estadios de sucesión secundaria y bosques primarios en el Parque Nacional Palo Verde, Costa Rica. *Revista Forestal Mesoamericana Kurú (Costa Rica)* **12**, 2215-2504 (2015).
148 Sanchez, A. Fidelity and Promiscuity in an Ant-Plant Mutualism: A Case Study of Triplaris and Pseudomyrmex. *PLOS ONE* **10**, e0143535, doi:10.1371/journal.pone.0143535 (2015).
149 Ramirez-Marcial, N., Camacho-Cruz, A. & Gonzalez-Espinosa, M. *Guía para la propagación de especies leñosas nativas de los Altos y montañas del Norte de Chiapas*. 41p (ECOSUR, 2003).
150 Fuel Puetate, M. A. *Estructura y composición florística de un bosque secundario en la microcuenca media del río Nangulví* BSc. Thesis thesis, Universidad Técnica del Norte, (2020).
151 Vasquez Quinticuari, P. K. *Restablecimiento de La sucesión ecológica secundaria en un biotopo de shapumba (Pteridium aquilinum (L.) Kuhn) en el caserío San Juan, región San Martín* BSc. Thesis thesis, Universidad Nacional Agraria de la Selva, (2020).
152 Pineda-Herrera, E., Valdez-Hernández, J. I. & López-López, M. Á. Fenología de Schizolobium parahyba y Vochysia guatemalensis en una selva alta perennifolia de Oaxaca, México. *Botanical Sciences* **90**, 185-193 (2012).

153 Healey, S. P. & Gara, R. I. The effect of a teak (Tectona grandis) plantation on the establishment of native species in an abandoned pasture in Costa Rica. *Forest Ecology and Management* **176**, 497-507, doi:https://doi.org/10.1016/S0378-1127(02)00235-9 (2003).
154 Sanmartín-Sierra, D. R., Angarita-Hernándeza, D. F. & Mercado-Gómez, J. D. Estructura y composición florística del bosque seco tropical de Sanguaré-Sucre (Colombia). *Ciencia en Desarrollo* **7**, 43-56 (2016).
155 Roig-Villariño, E. Y., Chacón Moreno, E., Capote López, R. P., Ferro Díaz, J. & Camejo, A. Floristic, structural and physiognomic recovery of tropical dry forest in la Jarreta, Península de Guanahacabibes. *Revista Anales de la Academia de Ciencias de Cuba* **4**, 1-17 (2014).
156 Ferreira, C. M., Finegan, B., Kanninen, M., Delgado, L. D. & Segura, M. Composición florística y estructura de bosques secundarios en el municipio de San Carlos, Nicaragua. *Revista Forestal Centroamericana* **38**, 44-50 (2002).

Appendix D. Summary of abundance per family and percentage (and cumulative) of individuals identified at the scientific level.

Plant Families	Abundance	% of individuals	Cumulative %
Fabaceae	34,843	24.6%	24.6%
Musaceae	31,826	22.5%	47.2%
Boraginaceae	16,761	11.9%	59.0%
Malvaceae	9,190	6.5%	65.5%
Bignoniaceae	6,414	4.5%	70.1%
Arecaceae	6,198	4.4%	74.4%
Meliaceae	5,131	3.6%	78.1%
Rutaceae	4,045	2.9%	80.9%
Burseraceae	3,563	2.5%	83.5%
Anacardiaceae	3,063	2.2%	85.6%
Myrtaceae	2,430	1.7%	87.3%
Lauraceae	2,332	1.6%	89.0%
Moraceae	1,917	1.4%	90.3%
Combretaceae	1,809	1.3%	91.6%
Fagaceae	1,466	1.0%	92.7%
Malpighiaceae	1,453	1.0%	93.7%
Sapindaceae	984	0.7%	94.4%
Sapotaceae	971	0.7%	95.1%
Urticaceae	807	0.6%	95.6%
Pinnaceae	665	0.5%	96.1%
Annonaceae	529	0.4%	96.5%
Rhamnaceae	444	0.3%	96.8%
Euphorbiaceae	423	0.3%	97.1%
Rubiaceae	363	0.3%	97.4%
Apocynaceae	277	0.2%	97.6%
Lamiaceae	254	0.2%	97.7%
Verbenaceae	250	0.2%	97.9%
Vochysiaceae	248	0.2%	98.1%
Piperaceae	202	0.1%	98.2%
Salicaceae	193	0.1%	98.4%
Calophyllaceae	170	0.1%	98.5%
Compositae	147	0.1%	98.6%
Bixaceae	139	0.1%	98.7%
Melastomataceae	135	0.1%	98.8%
Juglandaceae	132	0.1%	98.9%
Araliaceae	132	0.1%	99.0%
Solanaceae	116	0.1%	99.1%
Muntingiaceae	107	0.1%	99.1%
Olacaceae	104	0.1%	99.2%
Picramniaceae	104	0.1%	99.3%
Phyllanthaceae	94	0.1%	99.3%
Clethraceae	85	0.1%	99.4%
Asparagaceae	83	0.1%	99.5%
Hernandiaceae	66	0.0%	99.5%

Proteaceae	64	0.0%	99.6%
Chrysobalanaceae	61	0.0%	99.6%
Myristicaceae	61	0.0%	99.6%
Caricaceae	60	0.0%	99.7%
Altingiaceae	58	0.0%	99.7%
Vitaceae	41	0.0%	99.8%
Simaroubaceae	36	0.0%	99.8%
Schoepfiaceae	31	0.0%	99.8%
Polygonaceae	29	0.0%	99.8%
Hypericaceae	28	0.0%	99.8%
Rhizophoraceae	25	0.0%	99.9%
Cannabaceae	21	0.0%	99.9%
Primulaceae	20	0.0%	99.9%
Ebenaceae	19	0.0%	99.9%
Rosaceae	18	0.0%	99.9%
Nyctaginaceae	16	0.0%	99.9%
Ochnaceae	11	0.0%	99.9%
Pentaphylacaceae	11	0.0%	99.9%
Oxalidaceae	11	0.0%	99.9%
Dilleniaceae	10	0.0%	100.0%
Ulmaceae	8	0.0%	100.0%
Magnoliaceae	6	0.0%	100.0%
Adoxaceae	5	0.0%	100.0%
Asteraceae	5	0.0%	100.0%
Clusiaceae	5	0.0%	100.0%
Styracaceae	5	0.0%	100.0%
Cupressaceae	4	0.0%	100.0%
Actinidiaceae	4	0.0%	100.0%
Erythroxylaceae	4	0.0%	100.0%
Chloranthaceae	4	0.0%	100.0%
Lecythidaceae	3	0.0%	100.0%
Apoplanesia	3	0.0%	100.0%
Poaceae	1	0.0%	100.0%
Humiriaceae	1	0.0%	100.0%
Staphyleaceae	1	0.0%	100.0%
Capparaceae	1	0.0%	100.0%
Icacinaceae	1	0.0%	100.0%
Total species identified to species level	141,357	100.0%	

Species identified to common names and species non identified	6,898	4.7%
Total all individuals	148,255	

Appendix E: Supplementary figure of rarefaction curves by AFS

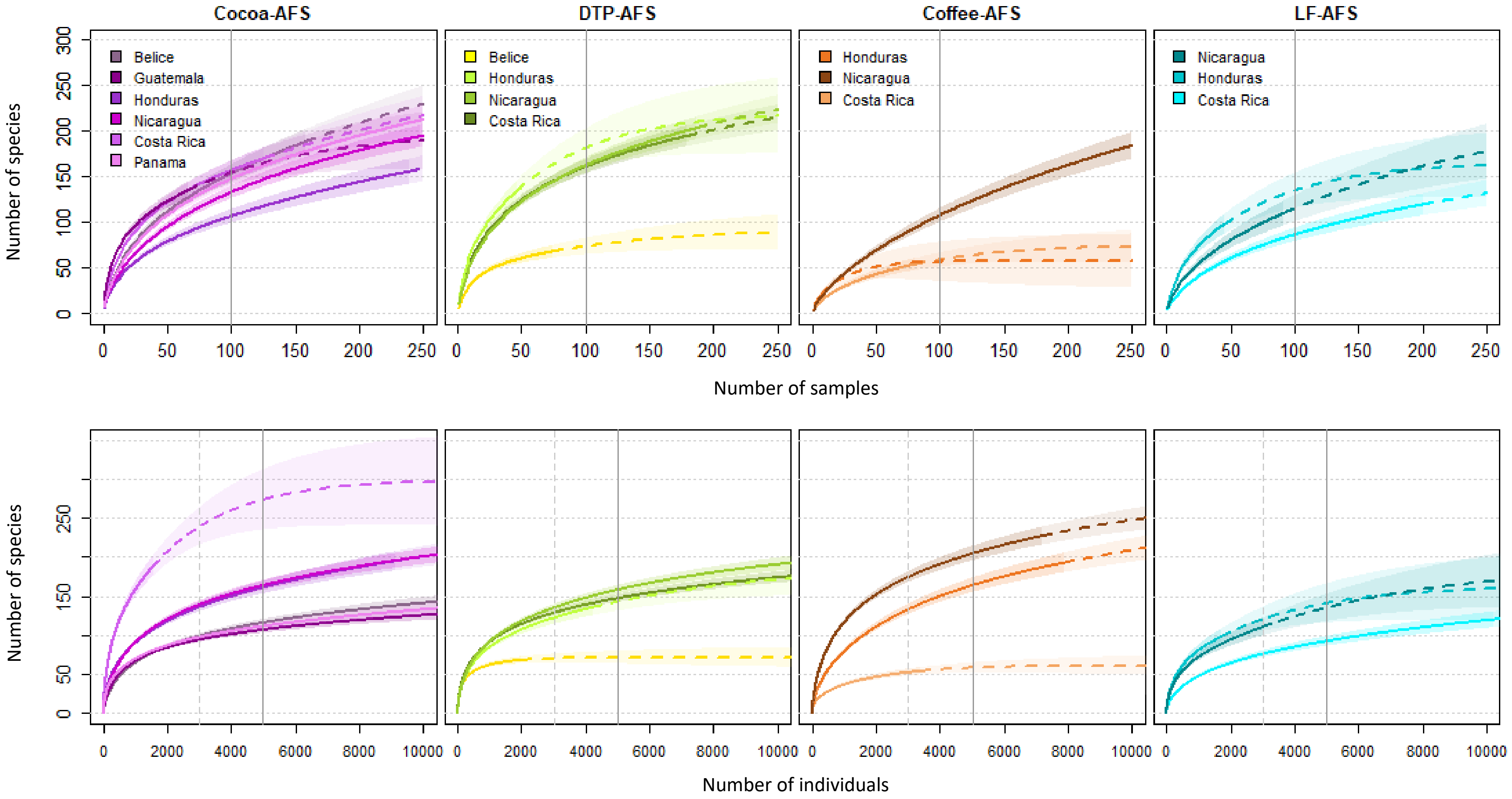

Figure E1. Rarefaction and extrapolation curves with Hill numbers (qD, for species richness where q = 0) per samples (sites), (upper panel) and per individuals (lower panel), for four agroforestry systems: COCOA-AFS (purple); COFFEE-AFS (brown); DTP (green) and LF (turquoise) across countries. Perpendicular lines represent the level at which comparisons for richness were made per samples: continuous grey line for comparisons at S100 = 100 samples and i5000= 5 000 individuals and dashed grey line for comparisons at S100 = 100 samples and i3000= 3 000 individuals, see methods for more details.
